# A Voltage-Gated Calcium Channel Regulates Lysosomal Fusion with Endosomes and Autophagosomes and Is Required for Neuronal Homeostasis

**DOI:** 10.1371/journal.pbio.1002103

**Published:** 2015-03-26

**Authors:** Xuejun Tian, Upasana Gala, Yongping Zhang, Weina Shang, Sonal Nagarkar Jaiswal, Alberto di Ronza, Manish Jaiswal, Shinya Yamamoto, Hector Sandoval, Lita Duraine, Marco Sardiello, Roy V. Sillitoe, Kartik Venkatachalam, Hengyu Fan, Hugo J. Bellen, Chao Tong

**Affiliations:** 1 Life Sciences Institute and Innovation Center for Cell Biology, Zhejiang University, Hangzhou, China; 2 Program in Developmental Biology, Baylor College of Medicine, Houston, Texas, United States of America; 3 Department of Molecular and Human Genetics, Baylor College of Medicine, Houston, Texas, United States of America; 4 Jan and Dan Duncan Neurological Research Institute, Texas Children’s Hospital, Houston, Texas, United States of America; 5 Howard Hughes Medical Institute, Baylor College of Medicine, Houston, Texas, United States of America; 6 Department of Pathology and Immunology, Department of Neuroscience, Baylor College of Medicine, Houston, Texas, United States of America; 7 Department of Integrative Biology and Pharmacology, University of Texas School of Medicine, Houston, Texas, United States of America; 8 Department of Neuroscience, Baylor College of Medicine, Houston, Texas, United States of America; Harvard Medical School, UNITED STATES

## Abstract

Autophagy helps deliver sequestered intracellular cargo to lysosomes for proteolytic degradation and thereby maintains cellular homeostasis by preventing accumulation of toxic substances in cells. In a forward mosaic screen in *Drosophila* designed to identify genes required for neuronal function and maintenance, we identified multiple *cacophony (cac)* mutant alleles. They exhibit an age-dependent accumulation of autophagic vacuoles (AVs) in photoreceptor terminals and eventually a degeneration of the terminals and surrounding glia. *cac* encodes an α1 subunit of a *Drosophila* voltage-gated calcium channel (VGCC) that is required for synaptic vesicle fusion with the plasma membrane and neurotransmitter release. Here, we show that *cac* mutant photoreceptor terminals accumulate AV-lysosomal fusion intermediates, suggesting that Cac is necessary for the fusion of AVs with lysosomes, a poorly defined process. Loss of another subunit of the VGCC, α2δ or *straightjacket (stj)*, causes phenotypes very similar to those caused by the loss of *cac*, indicating that the VGCC is required for AV-lysosomal fusion. The role of VGCC in AV-lysosomal fusion is evolutionarily conserved, as the loss of the mouse homologues, *Cacna1a* and *Cacna2d2*, also leads to autophagic defects in mice. Moreover, we find that CACNA1A is localized to the lysosomes and that loss of lysosomal *Cacna1a* in cerebellar cultured neurons leads to a failure of lysosomes to fuse with endosomes and autophagosomes. Finally, we show that the lysosomal CACNA1A but not the plasma-membrane resident CACNA1A is required for lysosomal fusion. In summary, we present a model in which the VGCC plays a role in autophagy by regulating the fusion of AVs with lysosomes through its calcium channel activity and hence functions in maintaining neuronal homeostasis.

## Abstract 2

A voltage-gated calcium channel required for neurotransmitter release also regulates the fusion of neuronal lysosomes with endosomes and autophagosomes, thereby helping to maintain cellular homeostasis.

## Author Summary

Autophagy is a cellular process used by cells to prevent the accumulation of toxic substances. It delivers misfolded proteins and damaged organelles by fusing autophagosomes—organelles formed by a double membrane that surrounds the “debris” to be eliminated—with lysosomes. How this fusion process is regulated during autophagy, however, remains to be established. Here, we analyze this process in flies and mice, and find that loss of different subunits of a specific type of Voltage Gated Calcium Channel (VGCC) leads to defects in lysosomal fusion with autophagosomes in neurons. It was already known that VGCCs control calcium entry at synaptic terminals to promote the fusion of synaptic vesicles with the plasma membrane, and that mutations in the subunits of VGCCs in humans cause neurological diseases. Our data indicate that defects in autophagy and lysosomal fusion are independent of defects in synaptic vesicle fusion and neurotransmitter release, and we show that a specific VGCC is present on lysosomal membranes where it is required for lysosomal fusion with endosomes and autophagosomes. These observations suggest that the fusion events required in autophagy rely on mechanisms similar to those that trigger the fusion of synaptic vesicles with the presynaptic membrane.

## Introduction

Autophagy is an evolutionarily conserved, lysosome-mediated degradation process required to maintain cellular homeostasis [1,2]. In eukaryotic cells, autophagy is a ubiquitous process that is important for several physiological processes. It occurs at a basal level in most cells to remove damaged organelles and is required for the turnover of long-lived proteins and other cellular macromolecules. Cellular quality control through autophagy is particularly relevant in long-lived neurons, as evidenced by autophagic malfunction in many human neurological disorders, including Alzheimer’s disease, Parkinson’s disease, Huntington’s disease, and amyotrophic lateral sclerosis (ALS) [3]. In both flies and mice, loss of autophagy-related genes leads to progressive neurodegeneration. It is still an open question whether neurons have their own tailored mechanism to regulate autophagy.

Autophagy is characterized by the formation of an isolation membrane that further elongates to form the double membrane autophagosome, which then fuses with the late endosomes and lysosomes [2]. Soluble N-ethylmaleimide-sensitive factor activating protein receptor (SNARE) proteins have been shown to be required for the fusion of autophagosomes with lysosomes. In yeast, the fusion of autophagosomes with vacuoles, the counterparts of lysosomes, involves the SNARE proteins Vti1 (Q04338.3), Ykt6 (CAA82040.1), Vam3 (CAA99304.1), and Vam7 (CAA96928.1) [4–7], but the latter two have no obvious homologues in metazoan cells. In *Drosophila*, the SNARE complex required for the fusion of autophagosomes with late endosomes and lysosomes consists of Syntaxin 17 (Syx17) (AGB94109.1), ubiSNAP (SNAP-29) (AAF47071.1), and Vamp7 (AHN56053.1) [8]. The requirement of these SNARE proteins for this fusion step is evolutionarily conserved as Vamp7, and Syntaxin 17 also play similar roles in mammalian cells [9]. Recent studies have shown that two pore channel (TPC), a lysosomal sodium channel, depolarizes lysosome membranes and promotes lysosome fusion upon PI(3,5)P2 stimulation or translocation of mammalian target of rapamycin (mTOR) away from the lysosome [10,11]. It is not established how the change in lysosome membrane potential coordinates the SNARE mediated fusion events.

In an unbiased genetic screen designed to isolate mutations that cause neurodegenerative phenotypes, we isolated many mutant alleles of *cacophony* (*cac*) (ID: 32158) that encode an α1 subunit of a *Drosophila* voltage-gated calcium channel (VGCC). VGCCs consist of multiple subunits, including the conducting pore forming subunit α1, and the accessory subunits α2δ, β, and γ [12]. The α1 subunit contains four internal repeats, each consisting of six transmembrane segments (S1–S6). The loop between transmembrane segments S5 and S6 of each repeat contains conserved domains for short segments 1 and 2 (ss1 and ss2). The calcium ion selectivity of the conducting pore is conferred by a conserved glutamate residue in the ss2 loop of each of the four internal repeats in the α1 subunits [13]. The α2δ subunit of VGCC consists of two disulfide-linked subunits, α2 and δ, derived from posttranslational cleavage of a single gene product [14,15]. In flies, a gene named *straitjacket* (*stj*) (ID: 36526) encodes the α2δ subunit which mediates the proper localization of Cac (P91645.3) at synapses [16]. Loss of *cac* is embryonic lethal in *Drosophila* and causes an almost complete loss of synaptic transmission [17,18]. *stj* mutants also exhibit a severe reduction in neurotransmitter release [16].

Mutations in human *Cacna1a* (ID: 773) and *Cacna2d2* (ID: 9254), the orthologs of *cac* and *stj* respectively, lead to severe neurological diseases, including episodic ataxia 2, familial hemiplegic migraine 1 (FHM1), absence epilepsy, progressive ataxia, and the polyglutamine disorder spinocerebellar ataxia 6 (SCA6) [19,20]. Mutations in two subunits of Ca_v_2.1 in mice, CACNA1A (AAW56205.1) and CACNA2D2 (Q6PHS9.1), also exhibit ataxia, epilepsy and neurodegeneration [21]. Aside from these spontaneous mutations, knock-in models of FHM1 and SCA6 have also been generated in mice. However, impairments in synaptic transmission do not underlie the mutant phenotypes observed in CACNA1A null mutant mice, and the molecular mechanisms underlying these diseases are still unclear [22]. Indeed, Jun et al. showed that in CACNA1A null mutant mice, excitatory synaptic transmission is largely unaffected because the N- and R-type VGCCs provide the calcium influx needed for synaptic vesicle (SV) fusion. However, these mice exhibit severe neurological deficits, implying that the P/Q-type VGCC plays other important roles than in synaptic transmission [22].

Here we show that, mutant alleles of *cac* and *stj* exhibit age-dependent autophagic defects in photoreceptor cells. We find that the role of the VGCC complex in neuronal autophagy is evolutionarily conserved as the loss of the mouse homologues, *Cacna1a* (ID: 12286) and *Cacna2d2* (ID: 56808), also result in autophagic defects in mice cerebella. We provide compelling evidence that the VGCC functions at the lysosomal fusion steps and that its role in autophagy is independent of its role in synaptic transmission. We further demonstrate that the α1 VGCC subunit CACNA1A is present on lysosomes, where it serves as a calcium channel required for lysosomal fusion with endosomes and autophagosomes. We propose that the VGCC in neurons regulates lysosomal fusion through its calcium channel activity on lysosomes.

## Results

### *cac* Is an Essential Gene to Maintain Neuronal Homeostasis in Photoreceptor Cells

**Fig 1:**
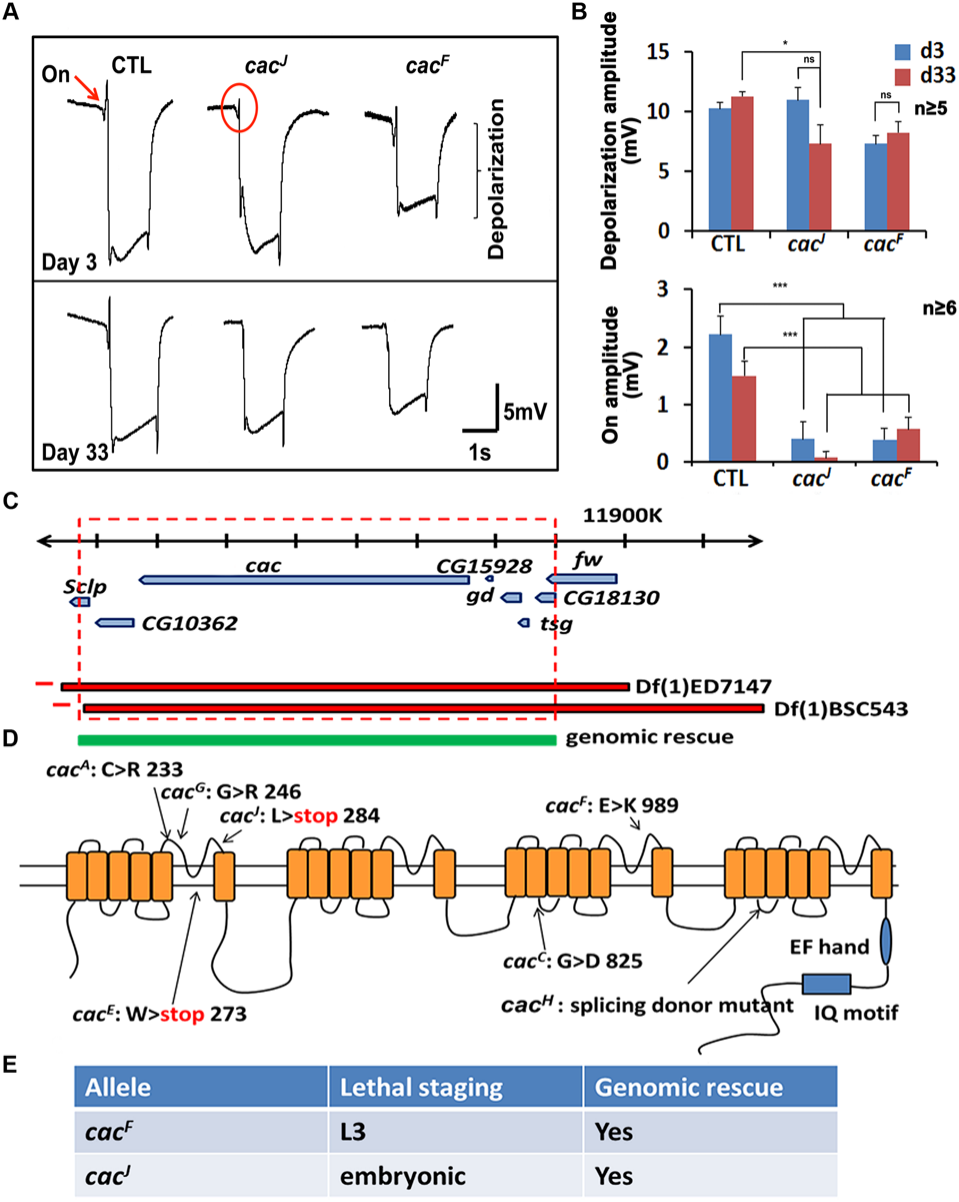
*cac* mutants show ERG defects. A. In response to a 1 s light pulse, *cac* alleles lack on transients at day 3 and day 33. B. Quantification of depolarization amplitudes (top) and on amplitudes (bottom). Data are presented as means ± standard error of the mean (SEM). C. The *cac* alleles identified failed to complement deficiencies Df(1)ED7417 and Df(1)BSC543. The lethality was rescued by 80 kb BAC clone CH321–60D21, narrowing the candidate region down to the area in the red box. We then crossed the *cac* alleles to existing alleles of *cac* and show that they fail to complement each other. D. Schematic representation of the molecular lesions of *cac* alleles. E. Lethal staging of *cac* alleles.

In order to identify essential genes on the X chromosome that are involved in neuronal homeostasis, we performed a forward genetic screen using ethyl methanesulfonate (EMS). We used the FLP/FRT system to induce homozygous mutant clones in the photoreceptor neurons of the otherwise heterozygous flies and performed electroretinograms (ERGs) on 3- and 33-days-old flies. Flies were exposed to a 1 s light pulse, and the electrophysiological responses were recorded. The amplitude of depolarization reflects photoreceptor activity and the on-off transients reflect pre- and post-synaptic connections. One of the complementation groups corresponds to *XE06*. The ERGs of these mutants exhibited a reduction of “on” transients in young and old animals (Fig. 1A and B), indicating a loss of synaptic transmission [23]. We mapped the mutations to *cac* using deficiency and duplication mapping (Fig. 1C). We then performed Sanger sequencing and identified seven different alleles (Fig. 1D): two early nonsense mutations, four missense mutations, and one splicing donor mutant. We selected two alleles for further characterization: *cac* ^*J*^ has an early nonsense mutation which is embryonic lethal and *cac* ^*F*^ has a missense mutation that destroys a key glutamate residue in the calcium ion selectivity loop and is third instar larval lethal (Fig. 1D and E). The lethality associated with both alleles is rescued with a transgene (Fig. 1E).

### *cac* Mutant Photoreceptor Terminals Degenerate and Exhibit Defects in Autophagy

**Fig 2:**
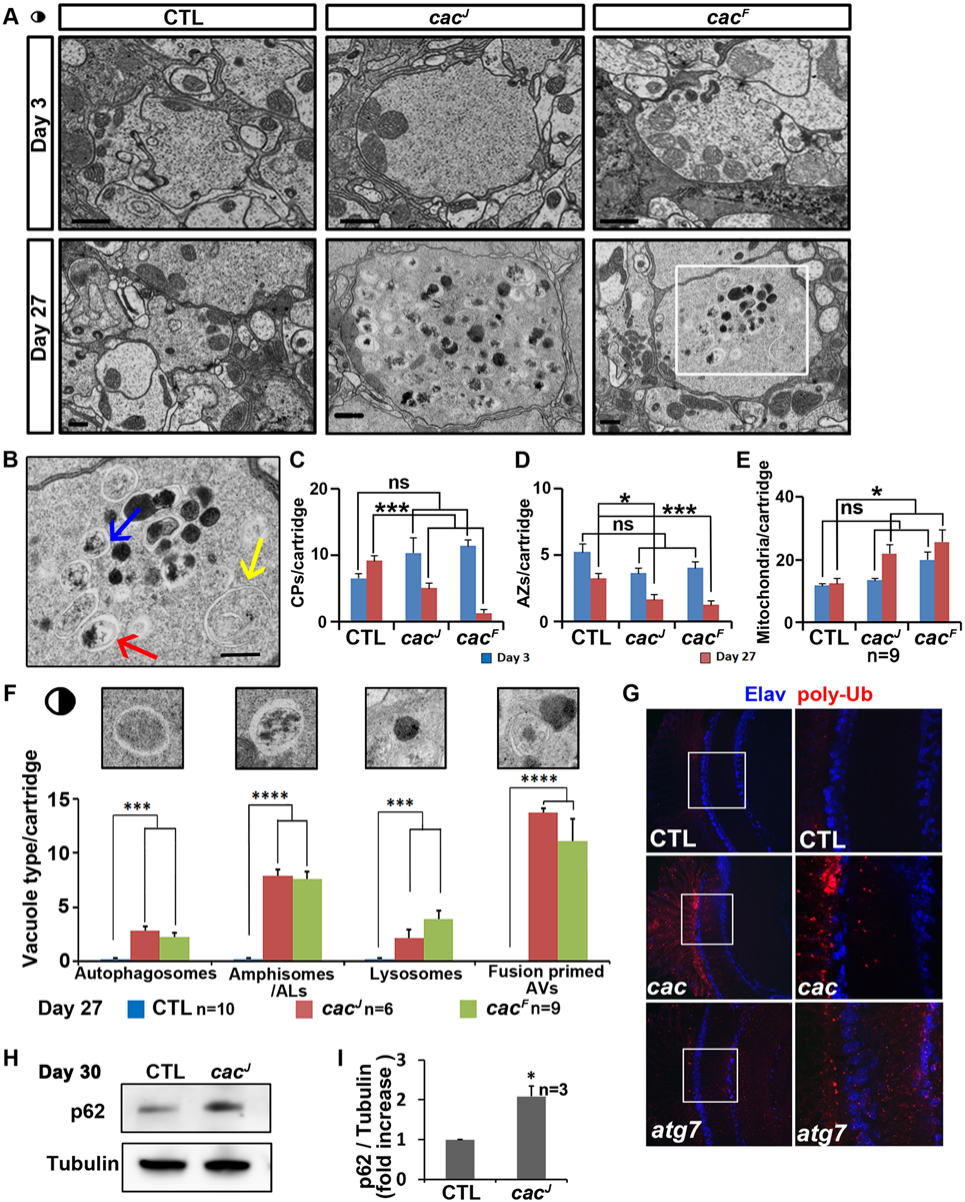
Aged *cac* mutant photoreceptor terminals show morphological defects and accumulate late stage AVs. A. TEM sections of 3- and 27-days-old photoreceptor terminals of flies. Terminals of 27-days-old flies show terminal expansion, loss of capitate projections (CPs) and active zones (AZs) and increased number of mitochondria per terminal. Scale bars, 500 nm. B. Inset from A representing the photoreceptor terminal boxed in white. Yellow arrow indicates double-membraned autophagosome, red indicates amphisome, and blue indicates two fusion-primed AVs. Scale bars, 500 nm. C-E. Quantification of CPs, AZs, and mitochondria per cartridge, respectively. Data are presented as means ± SEM. F. Quantification of the different AVs per cartridge. The mutant terminals accumulate late stage AVs and fusion-primed AVs (ns: not significant; *: *p* < 0.05; **: *p <* 0.01; ***: *p <* 0.001; ****: *p <* 0.0001). Data are presented as means ± SEM. G. 30-days old fly eye brain complexes stained with neuronal marker Elav (blue) and poly-Ub antibody (red). The right panel is the enlarged images of the boxed regions at the left side. H. The level of p62 is increased in *cac* ^*J*^ brain. I. Quantification of H. Data are presented as means ± SEM. (*: *p <* 0.05).

To examine whether the *cac* mutants have degenerative phenotypes in the eyes, we performed Transmission Electron Microscopy (TEM) on the mosaic eyes with most photoreceptor cells homozygous for *cac* mutant. As shown in Fig. 2A and S1 Fig, TEM of *cac* ^*J*^ and *cac* ^*F*^ photoreceptor terminals at day 3 show aberrantly expanded terminals that are more densely filled with SVs when compared to controls (CTL). As the flies age (day 27), the terminals expand further, and the cartridge structure in the lamina is lost (Fig. 2A and S1 Fig). In addition, the number of capitate projections (CPs) (Fig. 2C) and active zones (AZs) (Fig. 2D) decrease dramatically, whereas the number of mitochondria per terminal is increased (Fig. 2E). We also observed a significant accumulation of AVs in aged photoreceptor terminals, showing a progressive worsening of the phenotype (Fig. 2A, B and S1 Fig). The intermediate AVs, especially fusion-primed AVs (blue arrow in Fig. 2B) are greatly increased in aged mutants (Fig. 2F). This suggests that although *cac* mutant photoreceptor terminals form autophagosomes, there is a defect in autophagosomal maturation and fusion. The genomic fragment containing *cac* rescues both the morphology defects of photoreceptor terminals in the lamina and the accumulation of AVs in the photoreceptor terminals (S1 Fig).

Poly-ubiquitinated proteins are delivered to autophagosomes and degraded by lysosomes through AV-lysosomal fusion [24]. Therefore, they could serve as a marker to examine the autophagy flux. Indeed, impairment of autophagy has been shown to cause an increase in poly-ubiquitinated proteins in aged *Atg7* (ID: 37141) mutant flies [25]. To distinguish whether the accumulation of AVs in *cac* mutant flies is due to the blockage of autophagy at the late steps or the enhancement of autophagy induction, we measured the autophagic flux by monitoring poly-ubiquitinated proteins in *cac* mutant flies. We made mosaic flies with *cac* depleted in the eye, stained the eye-brain complexes of the aged *cac* flies with an anti-poly-Ubiquitin antibody, and compared the phenotype to the aged *Atg7* flies. As shown in Fig. 2G, *cac* mutant brains accumulate poly-ubiquitinated proteins similar to *Atg7* flies, supporting a defect in autophagy. To confirm that autophagic flux is reduced, we also examined protein levels of p62, one of the selective substrates for autophagy, using western blotting (Fig. 2H), in aged fly brains. We find an almost 2-fold increase in p62 in aged mutants, suggesting a reduction in autophagic flux (Fig. 2H and I). All these results, together with the TEM data, indicate that *cac* is required for autophagy.

### AV-lysosomal Fusion Defects in VGCC Mutants Are Not Related to Synaptic Transmission Defects

**Fig 3:**
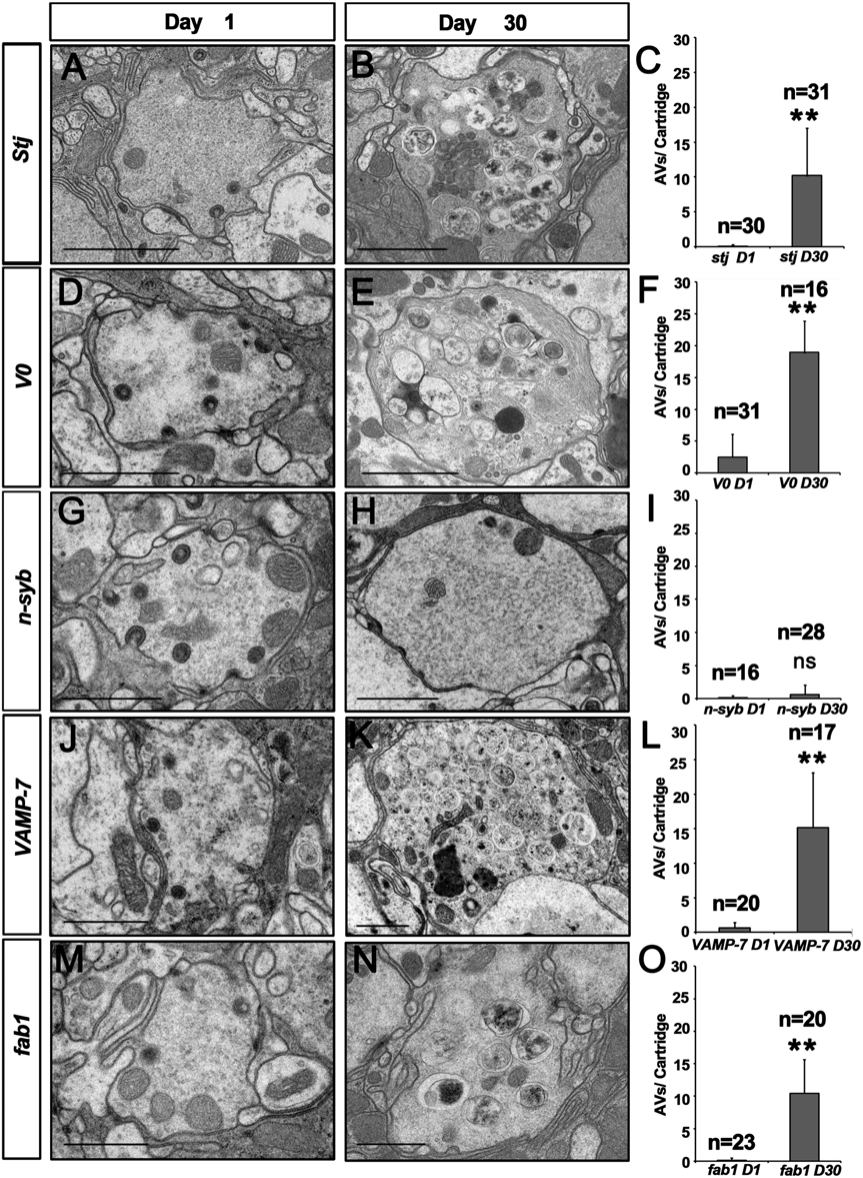
Autophagy defects observed in *cac* mutants are not due to defects in neurotransmitter release, but due to defects in lysosomal fusion. TEM sections of 1-day-old and 30-days-old photoreceptor terminals of flies raised in 12 h light/dark conditions. A, B, D, and E. *stj* and *Vha100–1 (V0)* mutant terminals accumulate AVs upon aging. G, H. *n-Syb* mutant photoreceptor terminals show increased SVs, but not a significant accumulation of AVs and fusion-primed AVs. J, K, M and N. *Vamp7* and *fab1* mutant photoreceptor terminals show the highest increase in AV accumulation. C, F, I, L, O. Quantification of the different AVs for each genotype (**: *p <* 0.01). Eyes from three animals for each genotype at each age were analyzed and the counted cartridge number were listed as “*n* = ” in the figure. Data are presented as means ± standard deviation (SD). Scale bars, 1 μm.

Cac regulates autophagy either through its VGCC channel activity or other activities independent of the calcium channel functions. To distinguish between these possibilities, we tested whether loss of other VGCC subunits leads to similar autophagy defects in flies. We examined the flies with a mutation for the α2δ subunit of VGCC encoded by the *stj* gene [16]. Loss of *stj* or *cac* causes very similar autophagic defects, a great accumulation of AVs, suggesting that the VGCC complex, not just Cac, is required for proper autophagy (Fig. 3A–C). We then examined several other mutants involved in lysosomal fusion and function. Loss of the Vacuolar H^+^ ATPase 100 kD subunit 1 (Vha100-1) (Q8IML5.1), a protein required for endosomal acidification [26], also results in accumulation of AVs in the photoreceptor terminals (Fig. 3D–F). Since *cac*, *stj*, and *Vha100–1* (ID: 43442)are all required for neurotransmitter release, and mutations in these genes result in SV accumulation in the photoreceptor terminals, it is possible that the autophagy defects we observed in these mutant terminals were a secondary effect of SV accumulation. However, loss of neuronal *synaptobrevin (n-Syb)* (ID: 38196), encoding a key regulator for neurotransmitter release, results in accumulation of SVs in the terminals [27] but does not lead to the autophagy phenotype (Fig. 3G–I). It indicates that loss of neurotransmitter release in *cac* and *stj* mutants is not causing autophagy defects per se. Moreover, loss of Vamp7 [28], a SNARE required for autophagosomal maturation and lysosomal fusion and Fab1 (O96838.2), a kinase required for autophagosomal-lysosomal fusion [29] cause similar AV accumulation phenotypes as *cac* and *stj* flies (Fig. 3J–O). These data confirm that Cac and Stj play a role in the AV-lysosomal fusion step of autophagy.

### The Role of the VGCC in Autophagy Is Conserved in Mice

**Fig 4:**
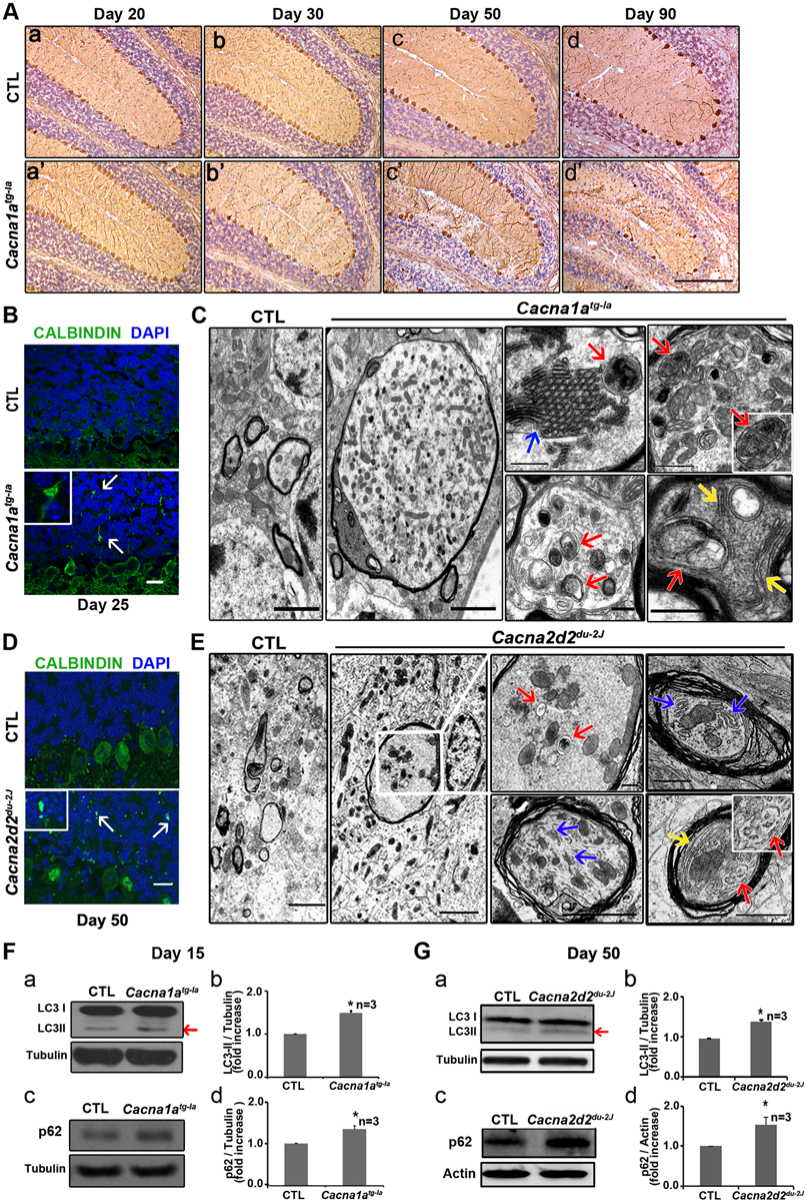
*Cacna1a* ^tg-la^ and *Cacna2d2* ^du-2J^ mice have autophagy defects. A. Calbindin and hematoxylin staining of mice cerebella indicates that *Cacna1a* ^*tg-la*^ mice have progressive PC and granule cell loss upon aging. Scale bar, 200 μm. B, D. Calbindin (green) and 4',6-diamidino-2-phenylindole (DAPI)(blue) staining of mice cerebellum indicates that there are swollen PC axons (arrows) in the granular layers of the *Cacna1a* ^*tg-la*^ mice cerebellum at day 25 (B) and the *Cacna2d2* ^*du-2J*^ mice at day 50 (D). Scale bar, 20 μm. C, E. The TEM of the axons at the granular layers of the cerebellum showing that many PC axons in *Cacna1a* ^*tg-la*^ and *Cacna2d2* ^*du-2J*^ mice are swollen and accumulated with numerous mitochondria, autophagosome/MVBs-like structures (red arrows), and various cytoplasmic vesicles or aberrant membrane structures, including stacks of cisternal membranes (blue arrows), and folded ER (yellow arrows). Scale bars for right two panels are 2 μm. Other scale bars are 500 nm. F, G. LC3II and p62 are slightly increased in 15-days-old *Cacna1a* ^*tg-la*^ mice and 50-days-old *Cacna2d2* ^*du-2J*^ mice. b, d are quantification of a and c. Data are presented as means ± SEM. (*: *p <* 0.05).

To determine if the role of Cac and Stj in autophagy is conserved in vertebrates, we obtained *leaner* (*Cacna1a* ^*tg-la*^) [30,31] and *ducky* (*Cacna2d2* ^*du-2J*^) mice [32,33] that carry mutations in orthologs of *cac* and *stj* respectively. *Leaner* mice have a splicing mutation in the *Cacna1a* locus [31], whereas *ducky* mice have a 2 bp deletion within the exon 9 of *Cacna2d2* [32]. These mice are deficient in neuronal autophagy and show striking similarities to the mice in which *Atg5* (ID: 11793) or *Atg7* (ID: 74244) is lost in neurons. The four mouse models exhibit motor defects, ataxia, reduced body size and weight, and smaller cerebella than the wild type (WT) littermates (S1 Table) [34–37]. In addition, these defects occur at similar ages in all these four mutant mice. *Cacna1a* ^*tg-la*^ mice have gradually narrowed granule cell layer and display a purkinje cell (PC) loss starting at day 30 that worsens gradually until there are almost no PCs visible in the anterior lobe at 3 months of age (Fig. 4A and S2 Fig). The degeneration of the *Cacna1a* ^*tg-la*^ mice cerebella resembles that of the *Atg5* and *Atg7* neuron specific knockout mice which show extensive PC loss by 2 months of age [34–37]. Moreover, all four mouse models exhibit swollen PC axons in the granule cell layers of the cerebella (S1 Table, Fig. 4B–E and S3 Fig).

To examine whether *Cacna1a* ^*tg-la*^ and *Cacna2d2* ^*du-2J*^ mice indeed suffer from autophagy defects, we performed ultrastructural studies on their cerebella using TEM. We observed many similarities between cerebella from these mutant mice and mice with neuronal autophagy defects. The swollen axons of both *Cacna1a* ^*tg-la*^ and *Cacna2d2* ^*du-2J*^ mice contain numerous abnormal-looking mitochondria, expanded membranes and endoplasmic reticulum (ER), expanded Golgi cisternal stacks and increased number of autophagosomes, multivesicular bodies (MVBs), and various cytoplasmic vesicles, a hallmark of lysosomal malfunction (S1 Table, Fig. 4C, E and S3 Fig). All of these phenotypes except for autophagosome accumulation have also been documented in mice with neuronal knockouts of *Atg7* [37], suggesting that *Cacna1a* ^*tg-la*^ and *Cacna2d2* ^*du-2J*^ mutant mice are also defective in autophagy. The difference of the autophagosome accumulation between the *Atg7* mice and the VGCC mutant mice probably is due to the fact that *Atg7* is required for autophagosome formation [38] whereas VGCC functions at the later fusion steps. We then probed the cerebellar lysates of both mutant mice with antibodies against autophagosomal protein LC3 and autophagy substrate p62 (Q64337.1) and observed a small but significant increase in the levels of both the LC3-II form of LC3 and p62 proteins, indicating that the autophagic flux is reduced in these mutant mice (Fig. 4F and G). Immunohistochemistry of *Cacna1a* ^*tg-la*^ Cerebella section also displayed increased levels of LC3 and p62 in the PC soma (S4 Fig), supporting a reduction in autophagic flux.

### CACNA1A Is Localized on the Lysosomes

**Fig 5:**
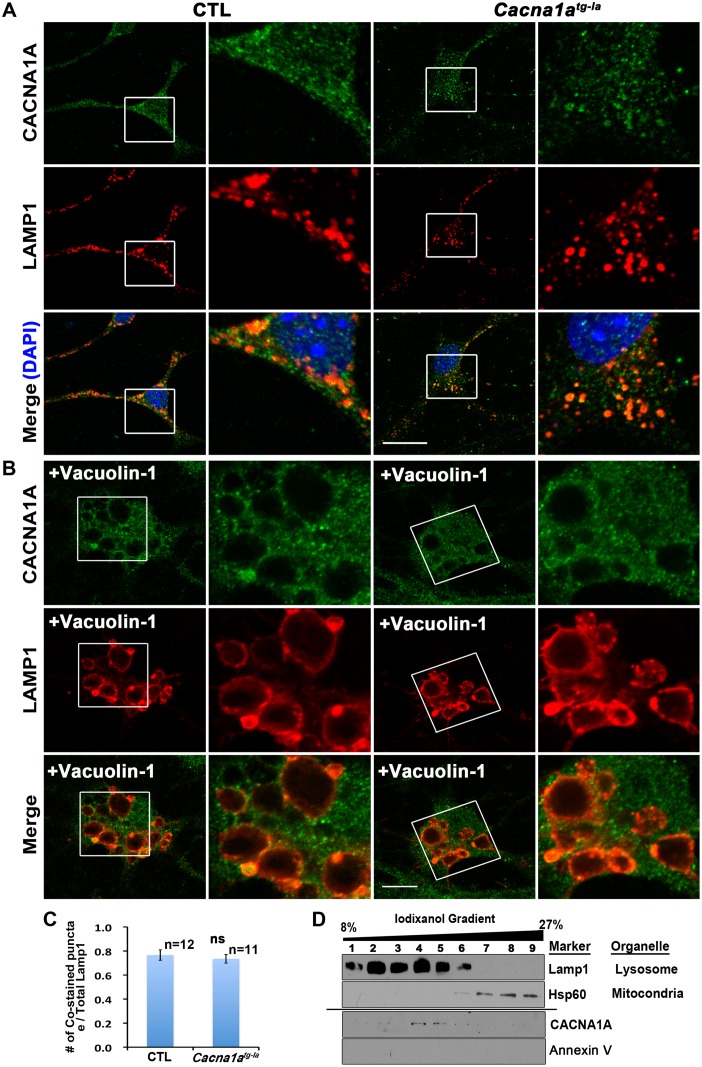
CACNA1A localizes to the lysosomes. A. CACNA1A co-localizes with LAMP1 in primary cerebellar cultures of both CTL and *Cacna1a* ^*tg-la*^ mutants. Scale bars, 20 μm. B. CACNA1A is present as punctae on the Vacuolin-1 enlarged LAMP1 positive lysosomes in primary cerebellar cultures of both CTL and *Cacna1a* ^*tg-la*^ mutants. Scale bars, 20 μm. C. Quantification of LAMP1 positive organelles that are also CACNA1A positive. Data are presented as means ± SD. (ns: not significant). D. CACNA1A is concentrated in the lysosomal fractions isolated from mice cerebella with iodixanol gradient.

The reduction in autophagy flux and the accumulation of MVBs and autophagosomes in mutant mice suggest that lysosomal fusion or degradation is compromised. It has been reported that CACNA1A not only localizes to the plasma membrane, but is also present in the neuronal cytosol [39]. To assess if CACNA1A affects lysosomal function by residing on lysosomes, we stained primary cultured cerebellar neurons with two different commercially available CACNA1A antibodies (Millipore and Abcam) and confirmed the specificity of the CACNA1A antibodies using peptide competition assays (S5 Fig). We observe a co-localization of CACNA1A with the lysosomal marker LAMP1 (P11438.2) both in WT and *Cacna1a* ^*tg-la*^ cerebellar neurons, showing that CACNA1A localizes to lysosomes (Fig. 5A, C and S6 Fig). We also stained the neurons with an early endosome marker (EEA1, Q8BL66.2) or ER marker (Calreticulin, P14211.1) in combination with the CACNA1A antibody and detect no obvious colocalization of CACNA1A with these two markers (S7 Fig and S8 Fig). To confirm lysosomal distribution of CACNA1A, we enlarged the lysosomes by pre-treating the primary neurons with Vacuolin-1 [40] and examined the distribution of CACNA1A with LAMP1 and CACNA1A antibodies. In both WT and *Cacna1a* ^*tg-la*^ primary cultured neurons, CACNA1A staining is present as punctae on the membrane of the enlarged lysosomes (Fig. 5B).

To provide independent biochemical evidence, we dissected cerebella from WT mice, and purified and separated lysosomes by iodixanol gradient and observe that full length CACNA1A is enriched in the lysosomal fractions (Fig. 5D). To exclude the possibility of plasma membrane contamination, we also probed the fractions with Annexin V antibody, and no signal was detected in the lysosomal fractions (Fig. 5D). In addition, we also extracted lysosomes from whole brains of WT mice using a subcellular fractionation protocol [41] and find that CACNA1A protein is present in the lysosomal fraction, which was verified with LAMP1 antibody (S9 Fig). The fainter CACNA1A band in the lysosomal lysate as compared to total brain lysate may be due to a much higher level of the protein on plasma membranes than lysosomes.

### *Cacna1a* Mutant Neurons Display Lysosomal Fusion Defects

**Fig 6:**
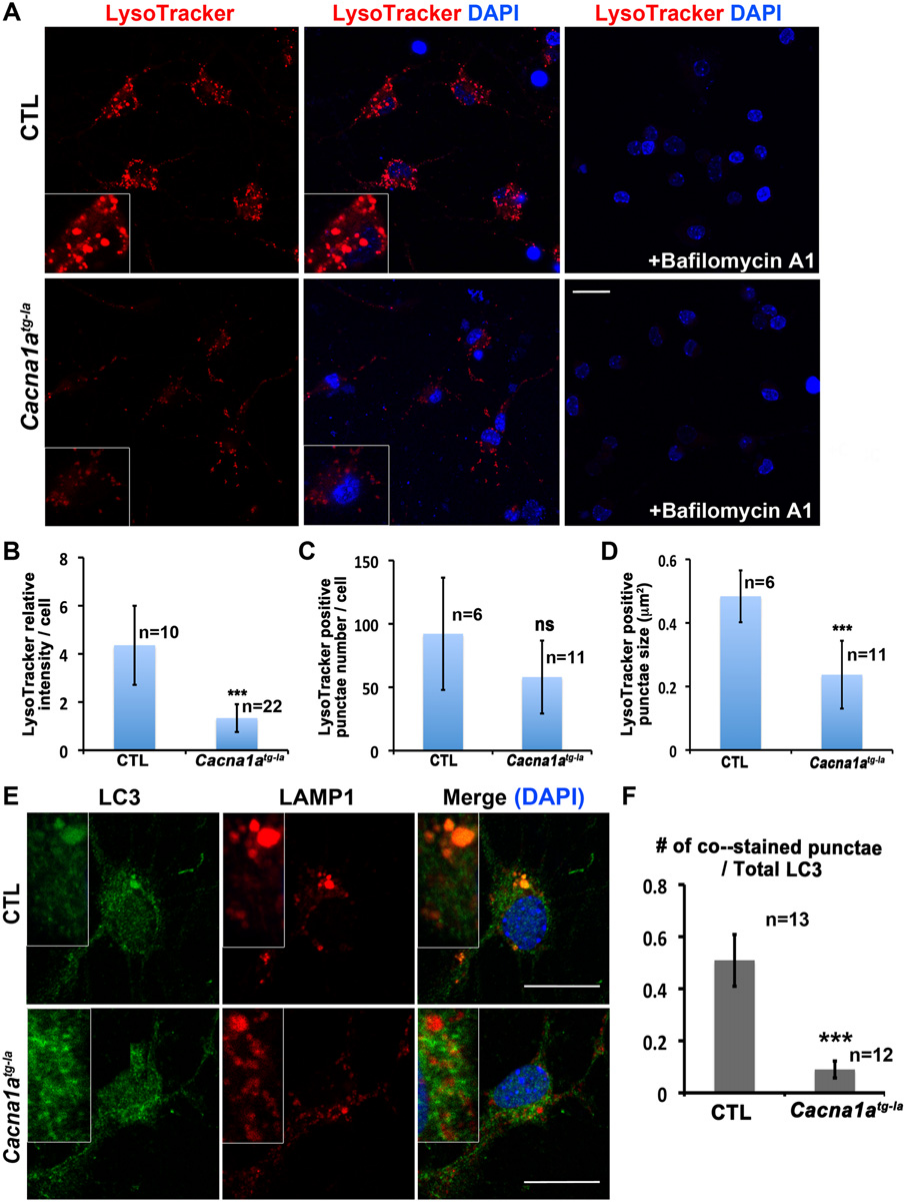
*Cacna1a* mutant neurons display lysosomal fusion defects. A. LysoTracker Red staining of the primary cerebella neurons from *Cacna1a* ^*tg-la*^ mice and WT controls. The control cells show big bright punctae, while the mutant cells have much dimmer and smaller punctae. B, C and D. Quantification of LysoTracker relative intensity (“n” represents the counted cell number), LysoTracker positive punctae number and size per cell (“n” represents the counted image number). E. Lysosomal marker LAMP1 co-localizes with autophagosome marker LC3 in CTL but not *Cacna1a* ^*tg-la*^ mutant cells. Scale bar, 20 μm. F. Quantification of LC3 and LAMP1 co-localization. Data are presented as means ± SD. (ns: not significant; ***: *p <* 0.001).

To assess whether lysosomal function is affected in the *Cacna1a* mutant cells, we isolated primary neurons from WT and *Cacna1a* mutant mice cerebella and stained the cells with LysoTracker Red DND-99. WT neurons show big and bright LysoTracker positive vesicles, and LysoTracker staining is almost completely abolished when the neurons are pre-treated with a lysosomal acidification inhibitor bafilomycin A1. As shown in Fig. 6A–D, *Cacna1a* mutant cells also display severely reduced LysoTracker staining, suggesting that lysosomes are impaired in *Cacna1a* mutant neurons.

In order to determine if *Cacna1a* deficient neurons exhibit defects in autophagosomal-lysosomal fusion, we co-stained primary cultured WT and *Cacna1a* mutant neurons with antibodies against a lysosomal marker (LAMP1) and an autophagosomal marker (LC3). In WT neurons, LC3 and LAMP1 co-localize, whereas little co-localization is observed in *Cacna1a* mutant cells (Fig. 6E and F), indicating that the fusion between AVs and lysosomes is affected in *Cacna1a* mutant neurons.

### Lysosomal CACNA1A, but Not Plasma Membrane Resident CACNA1A, Is Required for Lysosomal Fusion

**Fig 7:**
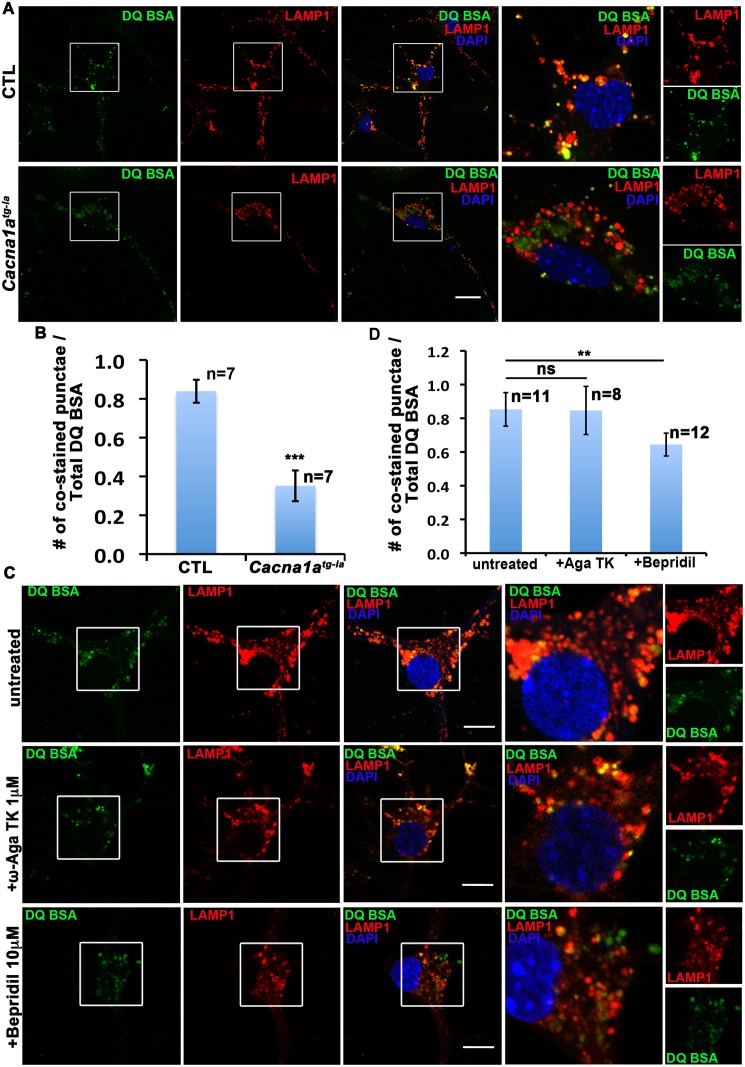
The calcium channel activity of CACNA1A on the lysosome, not the plasma-membrane is required for lysosomal fusion. A. DQ-BSA labeled late endosomes fuse with LAMP1 labeled lysosomes in CTL but not the mutant cells. Scale bar, 20 μm. B. Quantification of DQ-BSA and LAMP1 co-localization in CTL and *Cacna1a* ^*tg-la*^ cells. Data are presented as means ± SD. (ns: not significant; ***: *p <* 0.001) C. Bepridil but not ω-agatoxin TK (ω-Aga TK) treatment reduces lysosomal fusion in cultured cerebellar cells. DQ-BSA (Green) labeled late endosomes co-localize with LAMP1 (Red) labeled lysosomes with/without ω-Aga TK (1 µM) or Bepridil (10 µM) treatment. Scale bar, 20 μm. D. Quantification of DQ-BSA and LAMP1 co-localization with ω-Aga TK or Bepridil treatment. Data are presented as means ± SD. (ns: not significant; **: *p <* 0.001).

Lysosomes not only fuse with autophagosomes to degrade and recycle intracellular materials but also fuse with late endosomes to degrade and recycle membrane proteins and extracellular material [42]. To determine if lysosomal fusion with other organelles is compromised in *Cacna1a* mutant neurons, we labeled late endosomes and lysosomes with DQ-BSA. This fluorogenic proteolysis probe permits tracking of endocytic compartments after fluid-phase endocytosis [43]. As shown in Fig. 7A and C, LAMP1 co-localizes extensively with DQ-BSA in WT neurons, but not in *Cacna1a* mutant neurons. Indeed, numerous green and red punctae are observed in the mutant neurons, whereas most punctae are labeled yellow in WT neurons. These data show that lysosomes fail to fuse with late endosomes in *Cacna1a* cells.

Given that the primary role of a VGCC is its calcium channel activity and that a mis-sense mutation in the calcium ion selectivity pore in the fly homolog of *Cacna1a* causes lysosomal fusion defects similar to the loss of the gene, CACNA1A likely regulates lysosomal fusion through its calcium channel activity. Even though CACNA1A is present on lysosomes, the CACNA1A localized at the plasma membrane of synaptic terminals may be required for the calcium influx needed for lysosomal fusion in the cytosol. To rule out that CACNA1A activity on the plasma membrane is required for lysosomal fusion, we applied a P/Q-type calcium channel blocker ω-agatoxin TK at a saturating concentration (1 μM) [44,45] to primary cultured neurons and analyzed endosomal-lysosomal fusion with DQ-BSA. To first test whether ω-agatoxin TK could efficiently block the P/Q-type VGCC on the cell surface, we depolarized the neurons with high potassium chloride solution (90 mM) to activate VGCC, and recorded the calcium influx using a calcium indicator, Fluo 4-AM in the presence or absence of ω-agatoxin TK. ω-agatoxin TK greatly reduces calcium influx induced by the depolarization, indicating that the toxin is blocking depolarization-induced calcium entry via VGCCs (S10 Fig). However, we detected no obvious endo-lysosomal fusion defects in the WT neurons treated with the toxin (Fig. 7C and D). Since ω-agatoxin TK is not cell permeable, our data imply that the cell surface CACNA1A is not essential for lysosomal fusion. We then applied a cell-permeable calcium channel blocker Bepridil (10 µM) to the primary cultured neurons and analyzed endo-lysosomal fusion with DQ-BSA. We detected a significant reduction in colocalization between DQ-BSA and LAMP1 (Fig. 7C and D). A partial block of lysosomal fusion observed here may be due to insufficient block of the intracellular VGCC under current conditions. Taken together, our data suggested that the intracellular CACNA1A but not the cell surface CACNA1A is required for lysosomal fusion.

## Discussion

**Fig 8:**
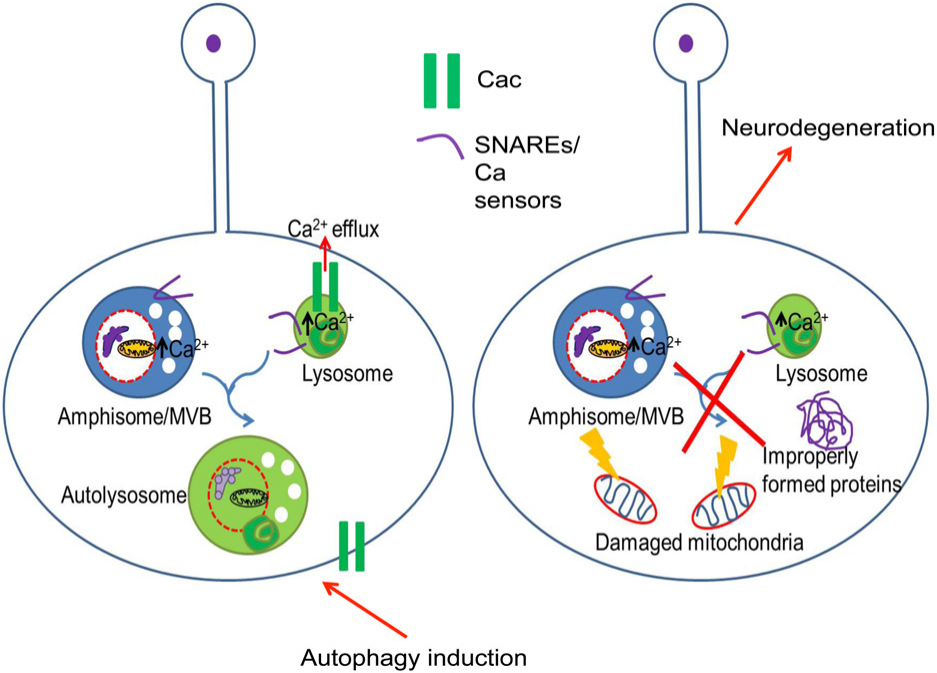
Cac is required on the lysosomes in neurons to promote lysosomal fusion. Cac and its accessory subunits are present on the lysosomes. Under normal conditions of activity requiring basal levels of autophagy, Cac allows for calcium efflux from endo/lysosomes to provide for SNARE-mediated fusion of autophagic vacuoles to lysosomes and subsequently efficient degradation of unwanted cellular material and to maintain neuronal homeostasis. In the absence of Cac, there is a block in the autophagic flux and lysosomal fusion, leading to an accumulation of fusion-primed autophagic vacuoles. This subsequently results in an accumulation of improperly formed proteins or damaged organelles, such as mitochondria in the neurons, and causes neurodegeneration.

Here, we show that CACNA1A is present on lysosomes and that it is required for endo-lysosomal fusion and autophagy (Fig. 8). Our work suggests that the VGCC regulates fusion of lysosomes with endosomes and AVs through its calcium channel activity on lysosomes. In the absence of VGCC subunits, as the neurons age and undergo basal autophagy, they accumulate AVs that are unable to fuse with lysosomes. Neurons then accumulate other damaged cellular organelles and misfolded proteins. Eventually this initiates a process of degeneration that mostly affects synapses and synaptic glial cells in fly eyes (Fig. 2A and S1 Fig). The latter phenotype is unlike most other neurodegenerative mutations, which cause a degeneration of rhabdomeres and cell body of the photoreceptors [46,47].

VGCCs have so far been implicated in the fusion of synaptic vesicles and dense core secretory vesicles with the plasma membrane as resident proteins of the plasma membrane [48]. In synapses, a sodium channel gates sodium upon an action potential that depolarizes the plasma membrane and promotes the activation of VGCC. The opening of the VGCC allows influx of extracellular calcium into the cytosol, which stimulates the assembly of the SNARE complex at the fusion site and subsequently promotes the fusion between neurotransmitter loaded vesicles and plasma membrane [49]. Although calcium and SNAREs have also been shown to be required for different steps of autophagy, a VGCC has not been implicated in autophagy previously. Our work suggests that the fusion events required in autophagy are not fundamentally different from those observed at presynaptic terminals for neurotransmitter release.

We show that CACNA1A is present on the lysosomal membrane in neurons. The cytosolic C terminal region of this protein contains a conserved YxxΦ motif across the different orthologs that is known to be required for lysosome targeting of some other lysosomal proteins (S1 Text) [50,51]. All these conserved motifs are remained in *Cacna1a* ^*tg-la*^. In addition, the CACNA2D2 protein is heavily glycosylated, similar to other lysosomal membrane proteins [15,52], which is known to protect lysosomal membrane proteins from proteolysis [53]. Thus it is likely that these characteristics might be responsible for the lysosomal localization of VGCC subunits, although this idea needs to be further investigated. It is not clear whether the delivery of VGCCs to lysosomes occurs by an indirect route via the plasma membrane or by a direct intracellular trafficking pathway. However, the route must ensure the correct topology of the channel on the lysosomal membrane so as to allow calcium to flow from the lysosome to the cytosol. The lysosomal ionic compositions are similar to the extracellular environment [10] and lysosomes are known to have high calcium content [54]. Studies have shown that the resting calcium concentration inside the lysosomal lumen of human macrophages is between 0.4–0.6 mM [55], a range that is much higher than the approximately 100 nM calcium concentration in the cytosol [56]. The 0.5 mM range of extracellular calcium concentration is sufficient to promote a robust calcium influx during synaptic transmission [57]. Hence, the luminal lysosomal calcium concentration is sufficient to induce calcium efflux from lysosomes through the VGCC to facilitate SNARE-mediated fusion of lysosomes with late endosomes and autophagosomes.

In addition to the requirement of high levels of resting luminal calcium concentration in the lysosomes, there should be a need for a depolarization of the lysosomal membrane to activate the VGCC. Recently, Cang et al. demonstrated that lysosomes are electrically excitable and contain a voltage-activated sodium channel NaV (formed by TPC1, Q9EQJ0.1) [58]. However, the role of this voltage depolarization in lysomes is unknown. Our findings imply that such a NaV-mediated depolarization may be able to activate the VGCCs to trigger Ca^2+^ efflux from lysosomes in neurons [10,11]. The activity of the sodium channels on the lysosome is triggered by PI(3,5)P2 stimulation or loss of lysosomal mTOR activity, and both are closely associated with autophagy and lysosomal fusion events [10,11,58]. Thus, the TPC proteins are well poised to act as the trigger to activate lysosomal VGCC and facilitate lysosomal fusion with autophagic or late endosomal organelles.

Another interesting question to consider is how the calcium efflux from the lysosomes through the VGCC triggers lysosomal fusion events. At the presynaptic plasma membrane, calcium regulates the fusion machinery through its binding to the C2 domains of synaptotagmin. As a calcium sensor, synaptotagmin interacts with SNAREs and phospholipids to facilitate fusion pore formation upon calcium entry [59]. Synaptotagmin VII (Syt7, Q9R0N7.1) is a calcium sensor that is present on lysosomes and has been shown to be required for lysosomal exocytosis during membrane repair [60]. It would therefore be interesting to test whether Syt7 or other calcium sensors participate in lysosomal fusion events.

In flies, *cac* is required in neurons because the neuronal specific expression of a transgene of *cac* can rescue the lethality and phenotypes associated with *cac* null mutant flies [18]. Mammalian P/Q-type VGCC subunits CACNA1A and CACNA2D2 are also expressed in neuronal tissues [31,32], and mutations in subunits in humans and mice mostly affect neurons. Hence, the requirement of the P/Q-type VGCC for lysosomal fusion might be specific to the neuronal system. Autophagy is a rather ubiquitous process that exists in all tissues. It is still an open question how lysosomal fusion is regulated in cells without P/Q-type VGCCs. Other calcium channels, such as transient receptor potential channels (TRPCs), may play a similar role in non-neuronal cells. Indeed, the TRPC homolog in yeast Yvc1 resides in the vacuole, a lysosome like organelle that can release Ca^2+^ in response to voltage changes. By contrast, the sole VGCC homolog in yeast localizes on the plasma membrane and is regulated by the stimuli that typically activate TRPC in animal cells [61]. The interchangeable regulation modes of TRPC and VGCC during evolution suggest they may play similar roles in certain conditions.

Our data also suggest a mechanism underlying the role of lysosomal dysfunction in the mouse model of human SCA6 [62,63]. SCA6 is a late-onset neurodegenerative disease caused by a polyglutamine tract expansion at the C terminal of CACNA1A. Unno et al. observed lysosomal involvement based on the accelerated neurodegeneration in SCA6 mice that were also lacking a key lysosomal cysteine protease in the cerebellum, Cathepsin B (P10605.2) [62]. However, they failed to detect autophagic defects in the SCA6 mouse model. This could be due to the low basal autophagy level in cerebella as evidenced by the low levels of LC3II in the cerebellar lysates (Fig. 4F and G) [64]. This may also explain why the AV accumulation in *Cacna1a* ^*tg-la*^ and *Cacna2d2* ^*du-2J*^ cerebella is not as dramatic as that in fly synapses (Fig. 4C, E and S3 Fig).

In summary, our work has uncovered an unexpected role of VGCC in AV- lysosomal fusion in neurons in helping to maintain cellular homeostasis and provides a new angle to our understanding of the pathology of *Cacna1a-* and *Cacna2d2*-related diseases in humans.

## Materials and Methods

### Ethics Statement

The experimental procedures using animals were in accordance with the National Institutes of Health Guide for the Care and Use of Laboratory Animals and approved by Zhejiang University Institutional Animal Care and Use Committee.

### Fly Genetics

*cac* mutants were isolated from an *ey-FLP* EMS screen as described previously [46]. The duplication and deficiency mapping were performed as described [65]. The genotypes of the fly strains generated in the paper are as following: Figs 1, 2 and S1 Fig CTL: *y w*, *iso FRT19A / P{w+} cl(1)FRT19A; eyFLP*. *cac* ^*J*^: *y w*, *cac* ^*J*^ *FRT19A/ P{w+} cl(1) FRT19A; eyFLP*. and *cac* ^*F*^: *y w*, *cac* ^*F*^ *FRT19A/ P{w+} cl(1) FRT19A; eyFLP*. S2 Fig rescue: *y w*, *cac* ^*J*^ *FRT19A/Y; Dp(1;3)DC131*. Fig. 2G: *Atg7* ^*d4*^. Fig. 3A and B: *y w*, *eyFLP; stj* ^*1*^ *FRT42D/ P{w+} cl(1) FRT42D* [16]. Fig. 3D and E: *y w*, *eyFLP; V100–1* ^*2*^ *FRT82B/ P{w+} cl(1) FRT82B* [66]. Fig. 3G and H: *y w*, *eyFLP; FRT syb* ^*ΔF33B*^ *FRT42D/ P{w+} cl(1) FRT42D* [67]. Fig. 3J and K: *y w*, *eyFLP; P(EP)VAMP7* ^*G7738*^ *FRT42D/ P{w+} cl(1) FRT42D*. Fig. 3M and N: *y w*, *eyFLP; fab1* ^*21*^ *FRT42D/ P{w+} cl(1) FRT42D* [29].

### Electroretinogram Recording

For ERG recording, y w *cac (lethal) FRT19A/FM7c, *Kr-Gal4*, *UAS-GFP* flies were crossed to *y w P{w+} cl(1) FRT19A/Dp(1;Y)y+; eyFLP* to generate flies with mutant clones in the eyes, and ERGs were performed as previously described [16]. At least five flies of each genotype were used for quantification.

### Immunohistochemistry for Fly Tissues

Dissected fly adult brains were fixed in PBS with 3.7% formaldehyde for 20 min, followed by washing with PBX (PBS + 0.4% Triton X-100) three times. The tissues were incubated with primary antibody overnight in 4°C followed by extensive washing and incubated with secondary antibody overnight at 4°C. After extensive washing, the samples were mounted in Vectashield (Vector Labs) followed by microscopy. Polyubiquitinylated conjugates antibody (FK1) was obtained from Enzo Life Sciences, 1:200 dilution was used. Elav antibody was obtained from Developmental Studies Hybridoma Bank and 1:100 dilution was used.

### Transmission Electron Microscopy for Fly Tissues

TEM was performed as described previously [68].

### Mouse Genetics and Culture

Heterozygous leaner mice with the control genotype, C57BL/6J: *tg* ^*la*^ */+* were originally obtained from The Jackson Laboratory in Bar Harbor, MA, United States. Male and female heterozygous leaner were mated to produce *tg* ^*la*^ */+* and homozygous *tg* ^*la*^ */tg* ^*la*^ offspring.

Male and female heterozygous ducky mice (*du* ^*2J*^ */+)* were originally obtained from The Jackson Laboratory and mated to each other to produce control (*+/+*) wild-type and homozygous mutant ducky (*du* ^*2J*^ */du* ^*2J*^) mice.

### Antibodies and Reagents

Rabbit polyclonal to LC3 antibody (Novus Biologicals, 1:200 dilution) and Mouse polyclonal to LC3 antibody (MBL, 1:50 dilution) were used for immunofluorescence studies and Rabbit pAb to LC3 (Novus Biologicals, 1:1,000 dilution) was used for immunoblotting. Two rabbit polyclonal to CACNA1A antibodies were purchased from Abcam (1:100 dilution) and Millipore (1:60 dilution) for immunofluorescence studies. Anti-CACNA1A antibody (Millipore, 1:1000 dilution) was used for immunoblotting. Anti-murine LAMP-1 (1D4B) mAb (1:1000 dilution for immunoblotting and 1:500 dilution for immunofluorescence studies) was purchased from Developmental Studies Hybridoma Bank. The mouse monoclonal antibody anti-p62 (1:500 dilution for immunohistochemistry and 1:1,000 dilution for immunoblotting) was from Abcam. Rabbit polyclonal antibody anti-Hsp60 (1:5,000 dilution) was from Epitomics. Rabbit mAb to tubulin (1:2,000 dilution) was from Cell Signaling, and rabbit pAb anti-Calbindin D-28K (1:500 dilution) was purchased from Millipore. Mouse anti-EEA1 mAb (1:1,000 dilution) was from MBL. Chicken pAb anti-Calreticulin (1:200 dilution) was from Abcam. DQ-BSA green and LysoTracker Red DND-99 were from Molecular Probes. Cytosine-β-D-arabinofuranoside were from Sigma. Bafilomycin A1 was from Tebu-Bio. ω-Agatonxin TK and Bepridil hydrochloride were purchased from Tocris Bioscience. Fluo 4-AM and Pluronic F-127 were from Dojindo Laboratories.

### Histology and Immunohistochemistry

Four percent paraformaldehyde-fixed, paraffin-embedded sections in 5 μm thickness were deparaffinized with xylene and washed with distilled water. Tissue sections were boiled for 20 min in 10 mM citrate buffer (pH 7.4). After antigen retrievals, all sections were washed in distilled water, treated with 0.3% (vol/vol) hydrogen peroxide to quench endogenous peroxide, and then incubated with normal goat serum for 30 min. Sections were incubated for 2 h at room temperature with primary antibodies. The primary antibodies were serially detected with the appropriate biotinylated anti-rabbit IgG (Vector), avidin-biotinylated-peroxidase complex (Vector), and, finally, developed with diaminobenzidine (Vector). The sections were washed, counterstained with hematoxylin, dehydrated, and mounted.

### Western Blot

Mice cerebella were homogenized in lysis buffer containing protease and phosphatase inhibitors. Protein concentration was determined using Bio-Rad protein assay reagent. Proteins were separated by SDS–PAGE, and transfer the protein onto a PVDF membrane. The membrane was blocked with 5% non-fat milk in TBST buffer and incubated with primary antibodies in 5% non-fat milk in TBST at room temperature for 1 h. Blots were incubated in goat anti-rabbit/mouse-HRP secondary antibody and diluted 1:2,500 in 5% non-fat milk/TBST for 1 h at room temperature. Blots were washed in TBST and then incubated with ECL reagent and exposed. Quantification of protein bands was done using the Image J software.

### Cerebella Culture

WT and *Cacna1a* ^*tg-la*^/ *Cacna1a* ^*tg-la*^ primary cerebella neurons were derived from P0-P2 pups. Cerebella were dissected from pups and individually digested with trypsin. Single cell suspensions obtained were plated on a poly-D-lysine-coated surface in Dulbecco's Modified Eagle's Medium (DMEM) supplemented with 10% (vol/vol) Fetal bovine serum (FBS) and 10% F12 Nutrient Mixture. 12 h after plating, culture medium was half replaced by serum-free neurobasal medium supplemented with B27 (Gibco) and L-Glutamine (Gibco). Mixed cultures were maintained at 37°C and 5% CO_2_. After 3 days in vitro (div), 5µM cytosine-β-D-arabinofuranoside was added to restrict glial cell growth. The cultures were used for experiments at 7 div–14 div.

### Electron Microscopy for Mice Tissue

The mice were anesthetized with 10% chloral hydrate (0.12 ml/10 g) and perfused with 0.9% NaCl, followed by a 100 ml mix of 1% paraformaldehyde and 1% glutaraldehyde, made in PBS (pH 7.4). After perfusion, cerebella were dissected and stored in fresh fixative overnight at 4°C. 0.5 to 1 mm sagittal sections of each cerebellum were postfixed with 2% osmium tetroxide for 2–3 h, dehydrated through an ascending series of ethanol and embedded in epon812. Ultrathin sections were cut, mounted on uncoated copper grids, stained with 2% uranyl acetate and 1% lead citrate for 12 min each. All the samples were observed using a Hitachi HT7700 electron microscope.

### Confocal Microscopy Methods

#### Immunofluorescent

Mice were fixed by perfusing 4°C 4% paraformaldehyde in PBS. The cerebella were dissected, further fixed in 4% paraformaldehyde in PBS for 2 h at 4°C, and frozen in OCT compound (Tissue-Tek). Frozen sections were cut and washed in PBS. After 4% paraformaldehyde fixation and blocking with 5% BSA in PBS/0.3% Triton X-100 for 30 min, frozen sections or cultured cells were incubated with primary antibodies for 1–2 h at room temperature. After washing with PBS, primary antibodies were detected with goat anti-mouse/rabbit/rat Alexa Fluor 488 (Jackson ImmunoResearch) and goat anti-mouse/rabbit/rat Cy3 (Jackson ImmunoResearch) for 1 h at room temperature. Sections were then washed and stained with 0.5 µg/ml DAPI in PBS for 5 min. For the antibody competition assay, the blocking peptide was incubated with the antibodies before applying the primary antibody to the culture cells. The following staining steps were the same as mentioned above.

#### Lysotracker staining

Growth media was exchanged for fresh, pre-warmed growth media containing 500 nM LysoTracker Red DND-99 labeling, and untreated cells or pretreated with 1 µM bafilomycin A1 were incubated at 37°C for 20 min. Cells were immediately placed on ice. After staining with 0.5 µg/ml DAPI in PBS for 2 min, cells were washed for 10 min in 4°C dye-free media.

#### DQ-BSA labeling

Cells were incubated with 25 µg/ml DQ-BSA Green in media at 37°C overnight, and chased for 2 h in normal culture media. For P/Q-type VGCC blocker treatment, ω-agatonxin TK at different concentrations (50 nM, 100 nM, 250 nM, 500 nM, and 1 µM) or Bepridil hydrochloride (10 µM) were added to the culture together with DQ-BSA. The following steps were the same as described above. Individual coverslips were washed twice with PBS, and fixed (4% PFA, 15 min) cells were then stained with anti-Lamp1 and DAPI.

All the mounted samples were examined and imaged on a confocal microscope LSM710 (Carl Zeiss) outfitted with a Plan‐Apochromat 63X 1.4NA oil immersion objective (Carl Zeiss). Data were collected using Carl Zeiss software ZEN 2010 and processed in Image J and Photoshop CS (Adobe).

### Calcium Imaging with Fluo 4-AM

Primary cultured cells were loaded with 2 µM Fluo-4 AM premixed with Pluronic F-127 in regular media for 30 min at 37ºC. Cells were washed in indicator-free media for three times and incubated for another 30 min to allow complete de-esterification of intracellular AM esters. Measurements were done at 37°C in Tyrode’s solution (NaCl 129 mM, KCl 5 mM, CaCl_2_ 2 mM, MgCl_2_ 1 mM, Glucose 35 mM, and HEPES 20 mM), and we added high potassium Tyrode’s solution (NaCl 5 mM, KCl 129 mM, CaCl_2_ 2 mM, MgCl_2_ 1 mM, Glucose 35 mM, and HEPES 20 mM) to a final concentration of 90 mM KCl for depolarization. Cell imaging was taken by high resolution living Cell system DeltaVision Elite.

### Lysosome Isolation

Fresh cerebella were dissected from mice that were starved overnight and killed the next morning. Then lysosome isolation by subcellular fractionation from the mice cerebella was performed with a lysosome isolation kit (Sigma-Aldrich) according to the manufacturer's manual. After a discontinuous iodixanol gradient centrifugation using Optima MAX-XP Benchtop Ultracentrifuge (Beckman Coulter) with MLS-50 rotor at 150,000 × g and 4°C for 4 h, the sample was divided into ten fractions (0.5 ml each) for further biochemical analyses.

### Lysosomal Subcellular Fractionation

Lysosomes were enriched by centrifugation from a pool of three independent mouse brains in a discontinuous Nycodenz density gradient, as described in [41], with modifications. Briefly, homogenate was prepared in assay buffer (0.25 M sucrose, pH 7.2) and centrifuged in succession at 4,800 × g, 5 min, and 17,000 × g, 10 min. The sediment of the second centrifugation was washed at 17,000 × g, 10 min, resuspended 1:1 vol/vol in 84.5% nycodenz, and placed on the bottom of an Ultraclear (Beckman) tube. On top, a discontinuous gradient of Nycodenz was constructed (layers from bottom to top were: 32.8%, 26.3%, and 19.8% Nycodenz). Centrifugation was for 1 h in an SW 40 Ti rotor (Beckman) at 141,000 × g. Lysosomes were collected from the 26.3/19.8 interface, diluted in 5–10 volumes of assay buffer and centrifuged at 37,000 × g, 15 min. Pellet was resuspended in 500 μl of assay buffer.

### Statistical Analysis

Data were analyzed by two-tailed unpaired Student’s *t* test. A *p*-value of <0.05 was considered statistically significant.

## Supporting Information

S1 Data Excel spreadsheets contain the numerical data for Fig. 1B, Fig. 2C, Fig. 2D, Fig. 2E, Fig. 2F, Fig. 2I, Fig. 3C, Fig. 3F, Fig. 3I, Fig. 3L, Fig. 3O, Fig. 4F, Fig. 4G, Fig. 5C, Fig. 6B, Fig. 6C, Fig. 6D, Fig. 6F, Fig. 7B, Fig. 7D, S1B Fig, S3 Fig, and S10 Fig, in separate sheets. (XLSX)

S1 Fig *Cac* mutants display synaptic terminal degeneration. A. These sections are taken at the lamina level with six photoreceptor terminals R1-R6 labelled in blue forming a cartridge around the interneurons L1-L2. Each cartridge bordered in yellow is surrounded by glial cells (gl). Aged mutant terminals are highly expanded, densely filled with SVs, lose their cartridge structure, and the glia surrounded cartridges cannot be identified. The autophagy and morphological defects are rescued by a genomic rescue construct of cac in the null mutant background. Scale bars, 500 nm. B. Quantification of mean terminal size. Blue columns represent day 3 and red columns represent day 27. Data are presented as means ± SEM. (***: *p <* 0.001).
(TIF)

S2 Fig *Cacna1a* ^tg-la^ mice show progressive neurodegeneration in cerebellum. Midsagittal cerebella sections were prepared from CTL or *Cacna1a* ^*tg-la*^ mice at different ages and stained with anti-Calbindin D-28K antibody to show the PC with hematoxylin counter stain. At day 30, PC loss was observed. At day 50, the front lobe of the cerebellum has more severe PC loss than the other part of the cerebellum. At day 90, most PCs are lost and the numbers of the granule cells also show very dramatic reduction. Scale bar, 1 mm.
(TIF)

S3 Fig *Cacna1a* ^tg-la^ and *Cacna2d2* ^du-2J^ mice exhibit swollen axons containing increased number of mitochondria and AV-MVBs. Statistics of the mean axonal area, numbers of mitochondria per axon and numbers of AV/MVBs per axon in day 25 *Cacna1a* ^*tg-la*^ mice and day 50 *Cacna2d2* ^*du-2J*^ mice at the granular layers of cerebellum.
(TIF)

S4 Fig *Cacna1a* ^tg-la^ mice cerebella accumulate LC3 and p62. Cerebella sections were prepared from CTL or *Cacna1a* ^*tg-la*^ mice mutant at day 15 and stained with anti-LC3 and anti-p62 antibodies respectively. Scale bar, 100 μm.
(TIF)

S5 Fig Antibody competition assay showing that CACNA1A antibodies staining is specific. A. The anti-CACNA1A antibody is from Millipore. B. The anti-CACNA1A antibody is from Abcam. Scale bar, 20 μm.
(TIF)

S6 Fig CACNA1A is present on lysosomes. The primary cerebella neurons from *Cacna1a* ^*tg-la*^ mice and wild-type controls were stained with a second CACNA1A antibody (green, Abcam) and anti-LAMP1 (red) antibody. Some of the CACNA1A is localized on the lysosomes. Scale bars, 20 μm.
(TIF)

S7 Fig CACNA1A is not present on early endosomes. The primary cerebellar neurons were stained with CACNA1A antibody (green) and early endosome marker EEA1 (red) antibody. Very few CACNA1A punctae co-localize with EEA1 labeled early endosomes. Scale bar, 20 μm.
(TIF)

S8 Fig CACNA1A is not present on endoplasmic reticulum. The primary cerebella neurons were stained with anti-CACNA1A antibody (red) and ER marker anti-Calreticulin (CARL, green) antibody. Very few CACNA1A punctae co-localize with CARL positive ER structures. Scale bar, 20 μm.
(TIF)

S9 Fig CACNA1A is enriched in the lysosomal extracts. The total brain lysate and the lysosomal enriched lysates were blotted with CACNA1A and LAMP1 antibodies. A band for CACNA1A protein was detected in the lysosomal lysate.
(TIF)

S10 Fig ω-agatoxin TK blocks the plasma membrane VGCC activity. The primary cultured cerebellar neurons are preloaded with membrane permeable calcium indicator Fluo 4-AM. Depolarization with high potassium chloride solution activates VGCCs on plasma membrane. In the presence of ω-Agatoxin, the calcium influx through VGCCs is greatly reduced.
(TIF)

S1 Table The phenotype comparisons of *Atg5/Atg7* neuronal knockouts and the VGCC mutant mice. (XLSX)

S1 Text The alignment of the protein sequences encoding by cac orthologs in different species to show the conserved potential lysosomal targeting sequences. A conserved YxxΦ motif is shown in a red box. Another two conserved motif similar but not identical to the lysosomal sorting signal shown in black boxes.
(DOCX)

## Abbreviations

ALS: amyotrophic lateral sclerosis
AV: autophagic vacuole
AZ: active zone
cac: cacophony
CALR: Calreticulin
CP: capitate projection
CTL: controls
DAPI: 4',6-diamidino-2-phenylindole
EMS: ethyl methanesulfonate
ER: endoplasmic reticulum
ERG: electroretinogram
FHM1: familial hemiplegic migraine 1
mTOR: mammalian target of rapamycin
MVB: multivesicular body
SD: standard deviation
SEM: standard error of the mean
PC: purkinje cell
n-Syb: neuronal synaptobrevin
SCA6: spinocerebellar ataxia 6
SNARE: soluble N-ethylmaleimide-sensitive factor activating protein receptor
ss1: short segments 1
ss2: short segments 2
stj: straightjacket
SV: synaptic vesicle
Syt7: Synaptotagmin VII
Syx17: Syntaxin 17
TEM: Transmission Electron Microscopy
TPC: two pore channel
TRPC: transient receptor potential channel
VGCC: voltage gated calcium channel
Vha100–1: the Vacuolar H+ ATPase 100kD subunit 1
WT: wild type
