# Supplementary figures and images for: A Voltage-Gated Calcium Channel Regulates Lysosomal Fusion with Endosomes and Autophagosomes and Is Required for Neuronal Homeostasis
Source: PLoS Biol. 2015 Mar 26;13(3):e1002103. doi: 10.1371/journal.pbio.1002103 (PMC4374850; doi:10.1371/journal.pbio.1002103)

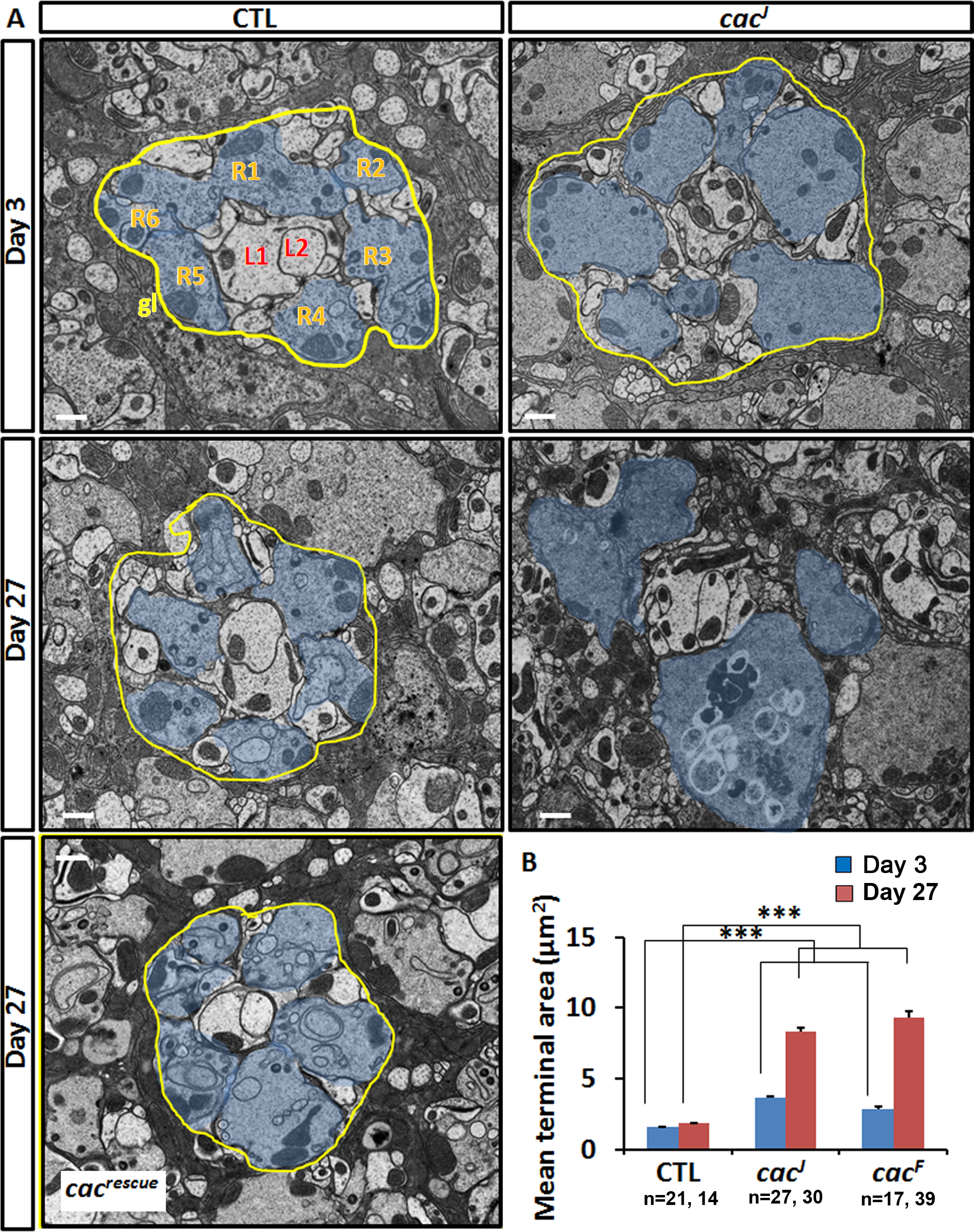

Supplement: S1 Fig — A. These sections are taken at the lamina level with six photoreceptor terminals R1-R6 labelled in blue forming a cartridge around the interneurons L1-L2. Each cartridge bordered in yellow is surrounded by glial cells (gl). Aged mutant terminals are highly expanded, densely filled with SVs, lose their cartridge structure, and the glia surrounded cartridges cannot be identified. The autophagy and morphological defects are rescued by a genomic rescue construct of cac in the null mutant background. Scale bars, 500 nm. B. Quantification of mean terminal size. Blue columns represent day 3 and red columns represent day 27. Data are presented as means ± SEM. (***: p < 0.001). (TIF) [file pbio.1002103.s002.tif]

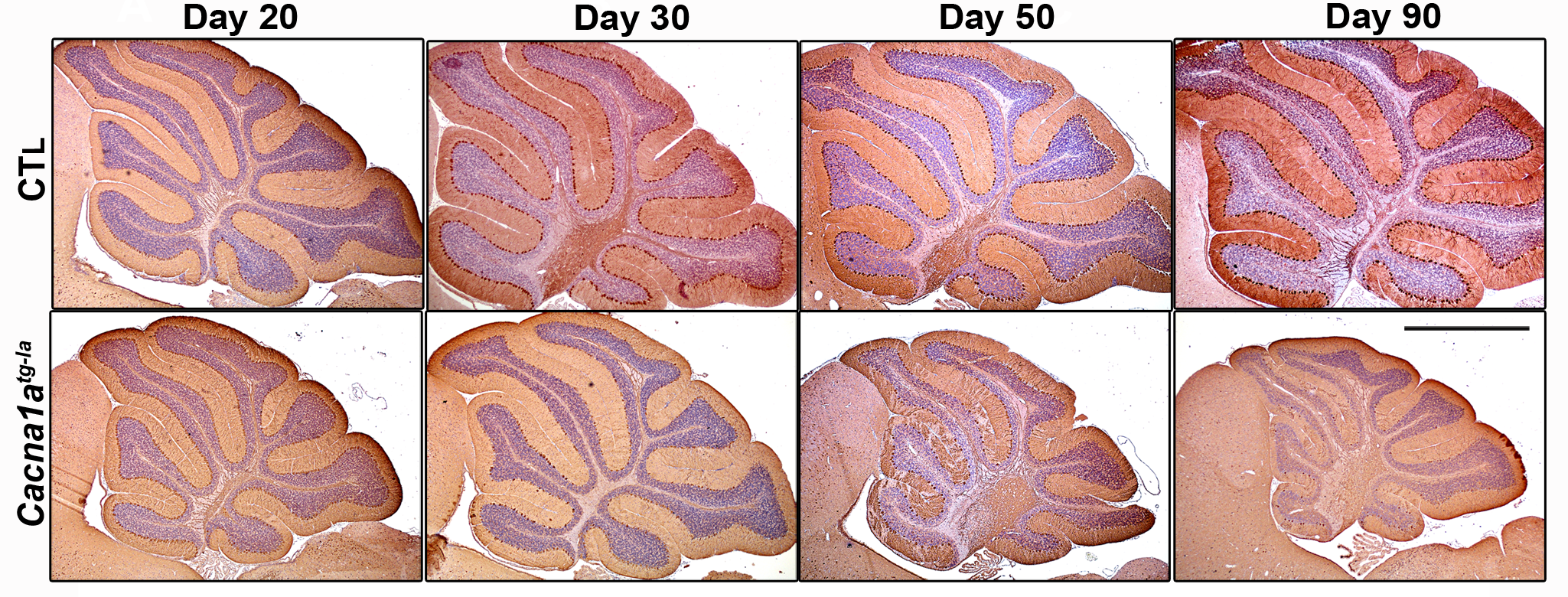

Supplement: S2 Fig — Midsagittal cerebella sections were prepared from CTL or Cacna1a tg-la mice at different ages and stained with anti-Calbindin D-28K antibody to show the PC with hematoxylin counter stain. At day 30, PC loss was observed. At day 50, the front lobe of the cerebellum has more severe PC loss than the other part of the cerebellum. At day 90, most PCs are lost and the numbers of the granule cells also show very dramatic reduction. Scale bar, 1 mm. (TIF) [file pbio.1002103.s003.tif]

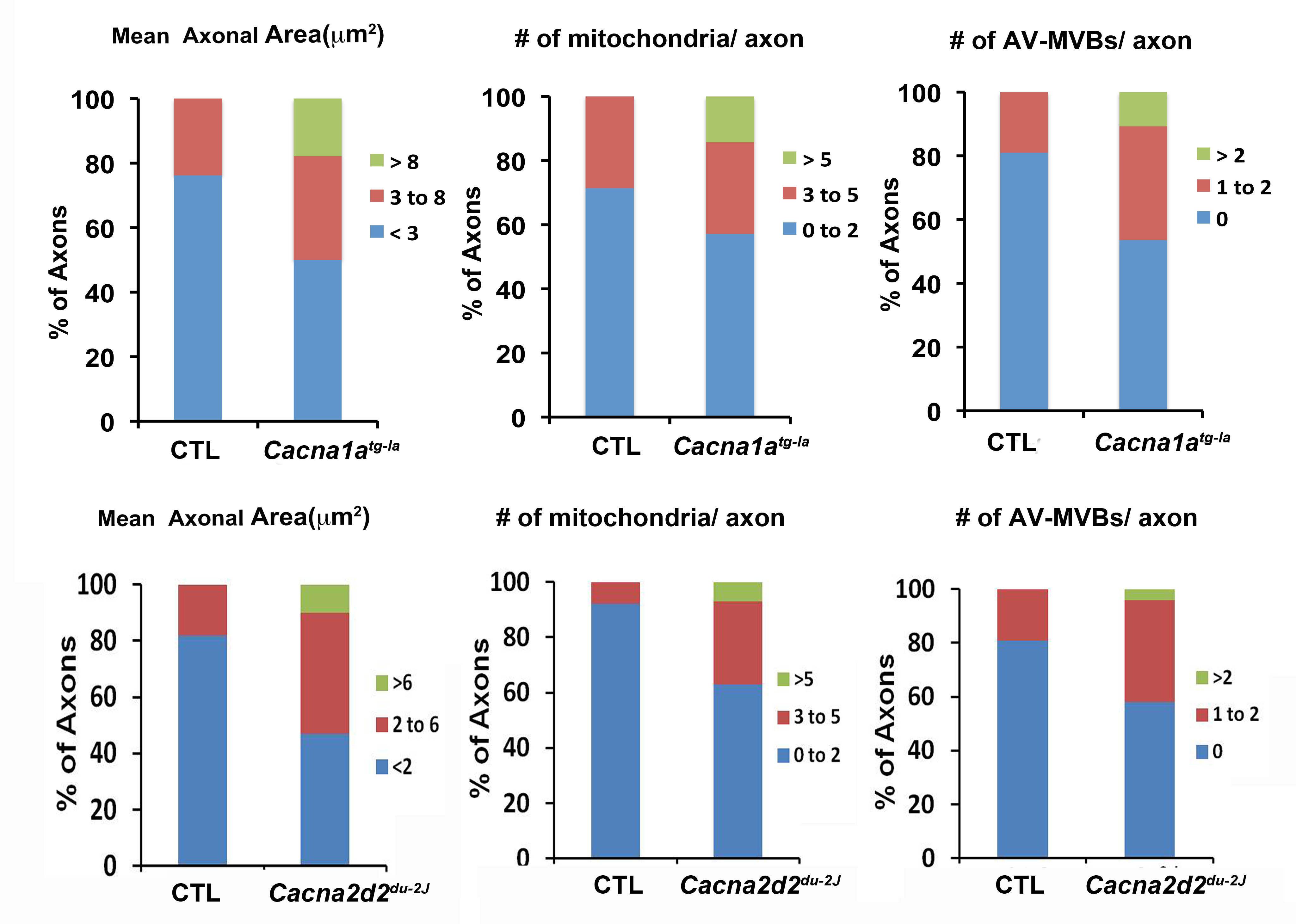

Supplement: S3 Fig — Statistics of the mean axonal area, numbers of mitochondria per axon and numbers of AV/MVBs per axon in day 25 Cacna1a tg-la mice and day 50 Cacna2d2 du-2J mice at the granular layers of cerebellum. (TIF) [file pbio.1002103.s004.tif]

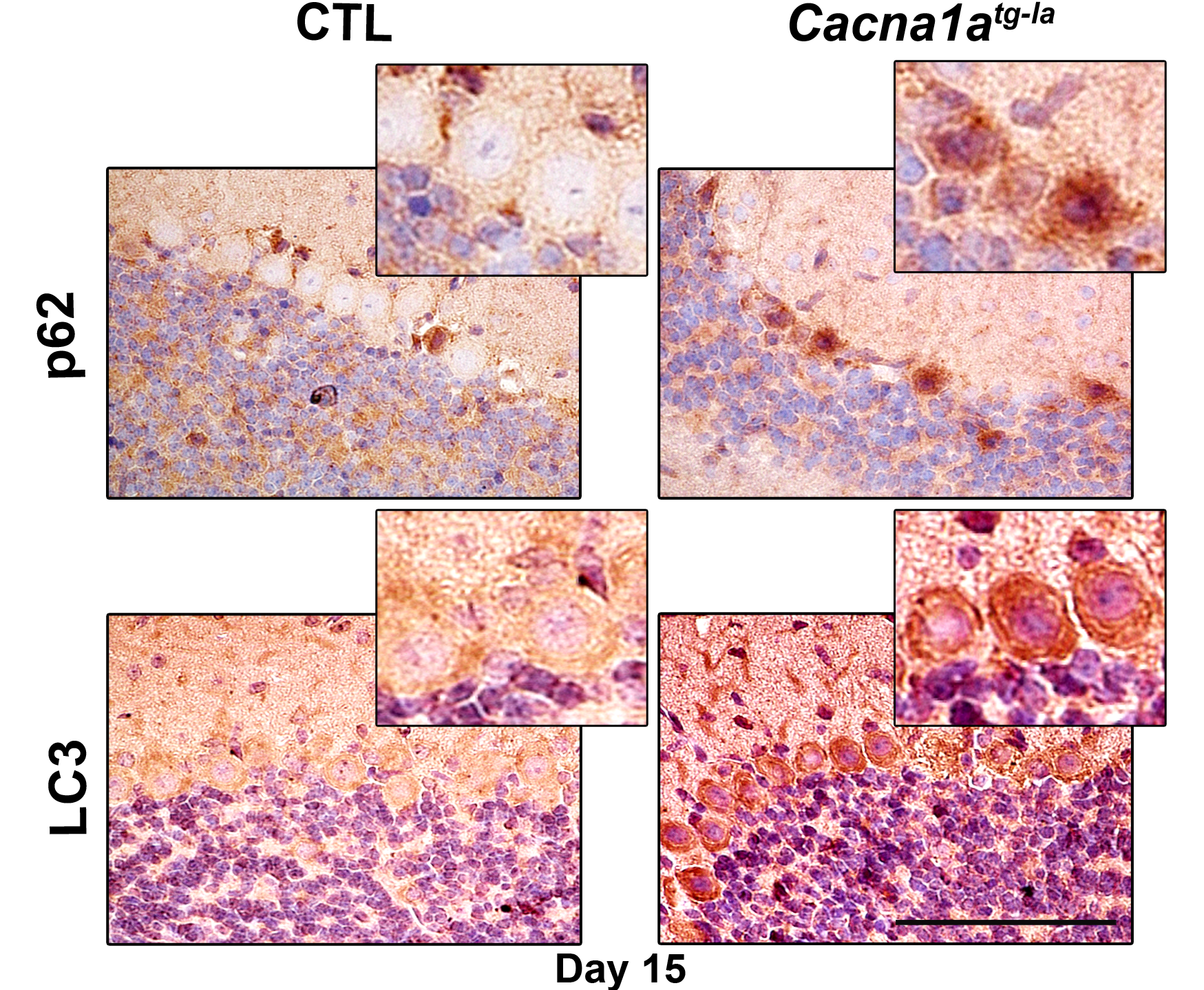

Supplement: S4 Fig — Cerebella sections were prepared from CTL or Cacna1a tg-la mice mutant at day 15 and stained with anti-LC3 and anti-p62 antibodies respectively. Scale bar, 100 μm. (TIF) [file pbio.1002103.s005.tif]

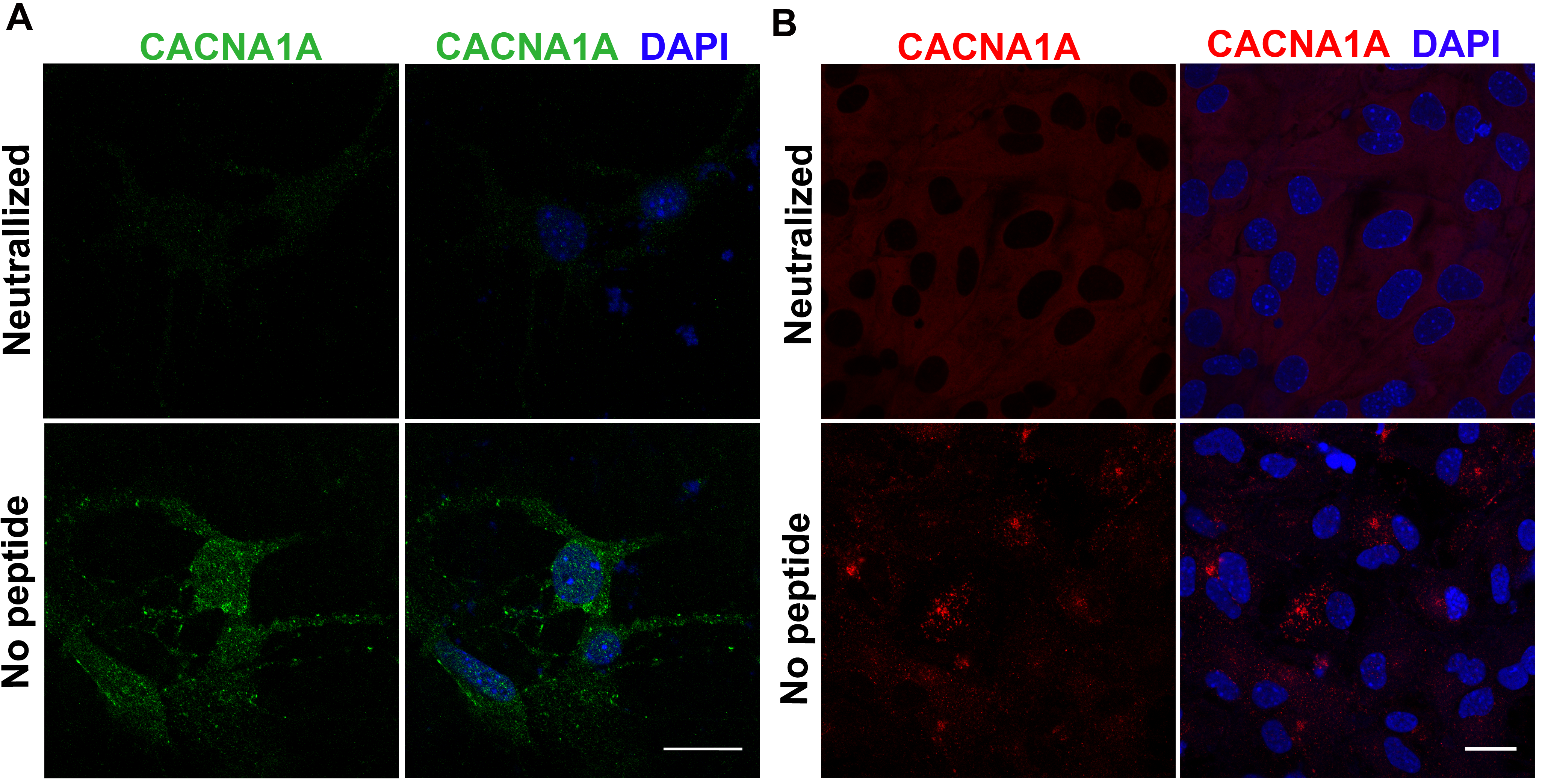

Supplement: S5 Fig — A. The anti-CACNA1A antibody is from Millipore. B. The anti-CACNA1A antibody is from Abcam. Scale bar, 20 μm. (TIF) [file pbio.1002103.s006.tif]

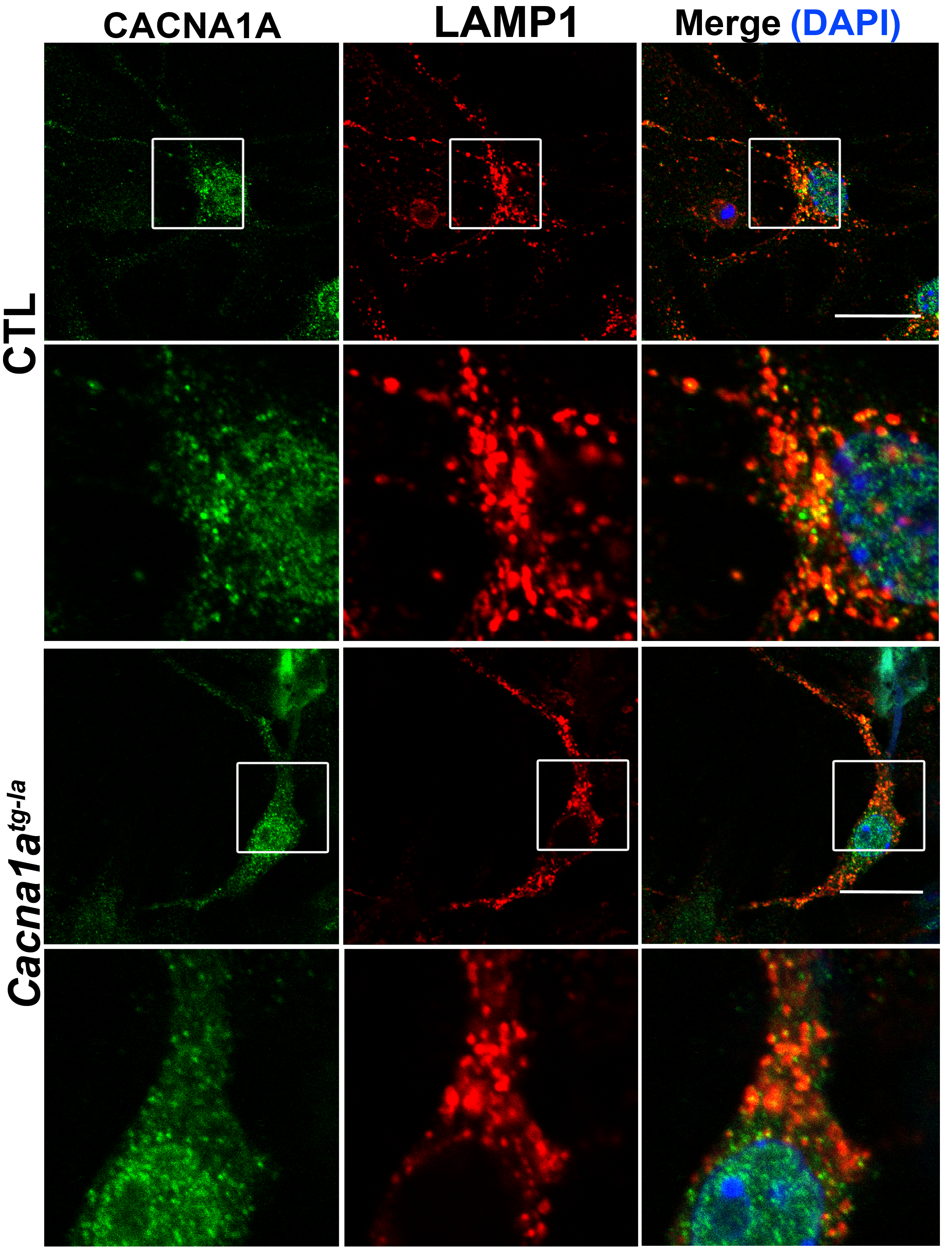

Supplement: S6 Fig — The primary cerebella neurons from Cacna1a tg-la mice and wild-type controls were stained with a second CACNA1A antibody (green, Abcam) and anti-LAMP1 (red) antibody. Some of the CACNA1A is localized on the lysosomes. Scale bars, 20 μm. (TIF) [file pbio.1002103.s007.tif]

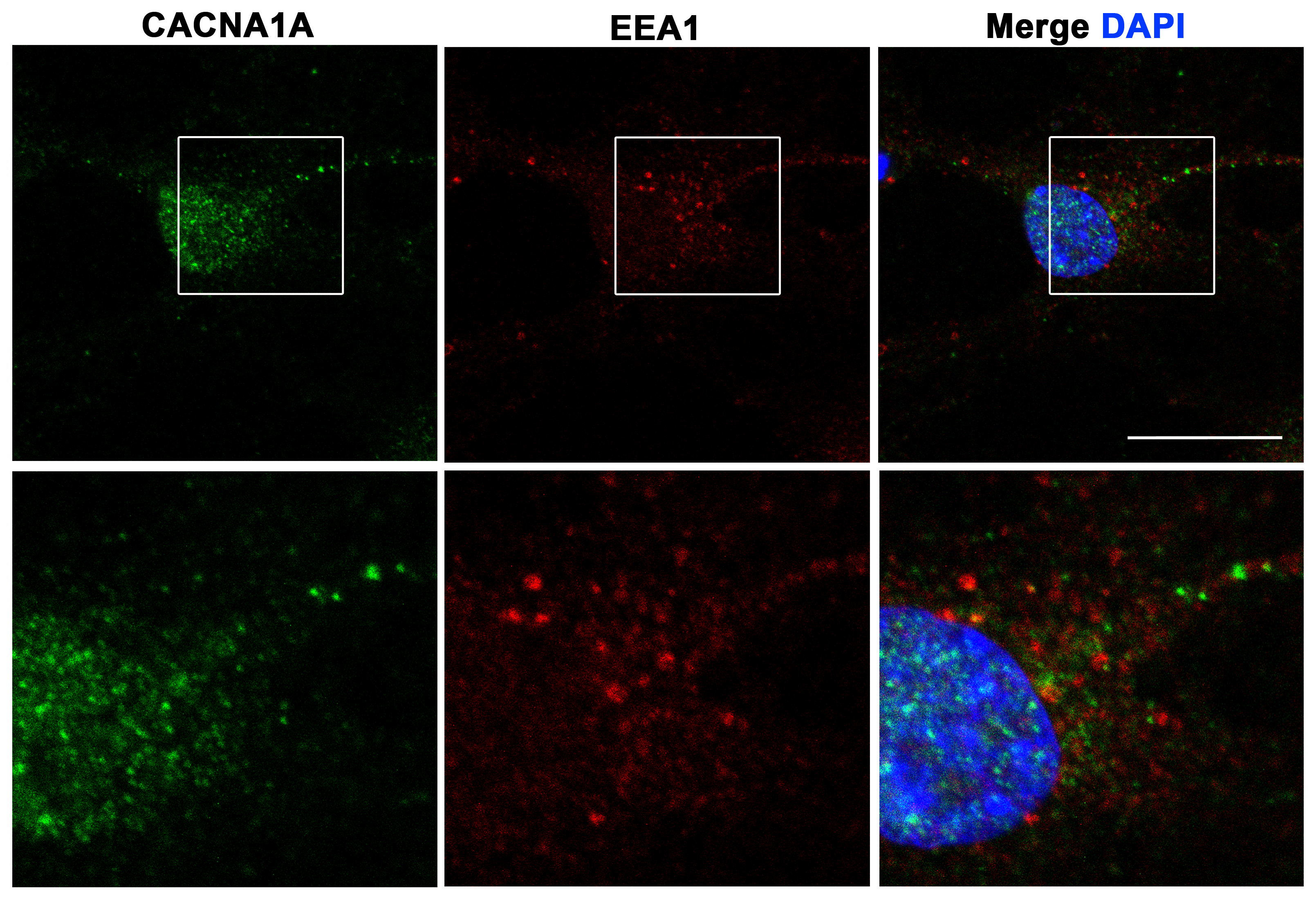

Supplement: S7 Fig — The primary cerebellar neurons were stained with CACNA1A antibody (green) and early endosome marker EEA1 (red) antibody. Very few CACNA1A punctae co-localize with EEA1 labeled early endosomes. Scale bar, 20 μm. (TIF) [file pbio.1002103.s008.tif]

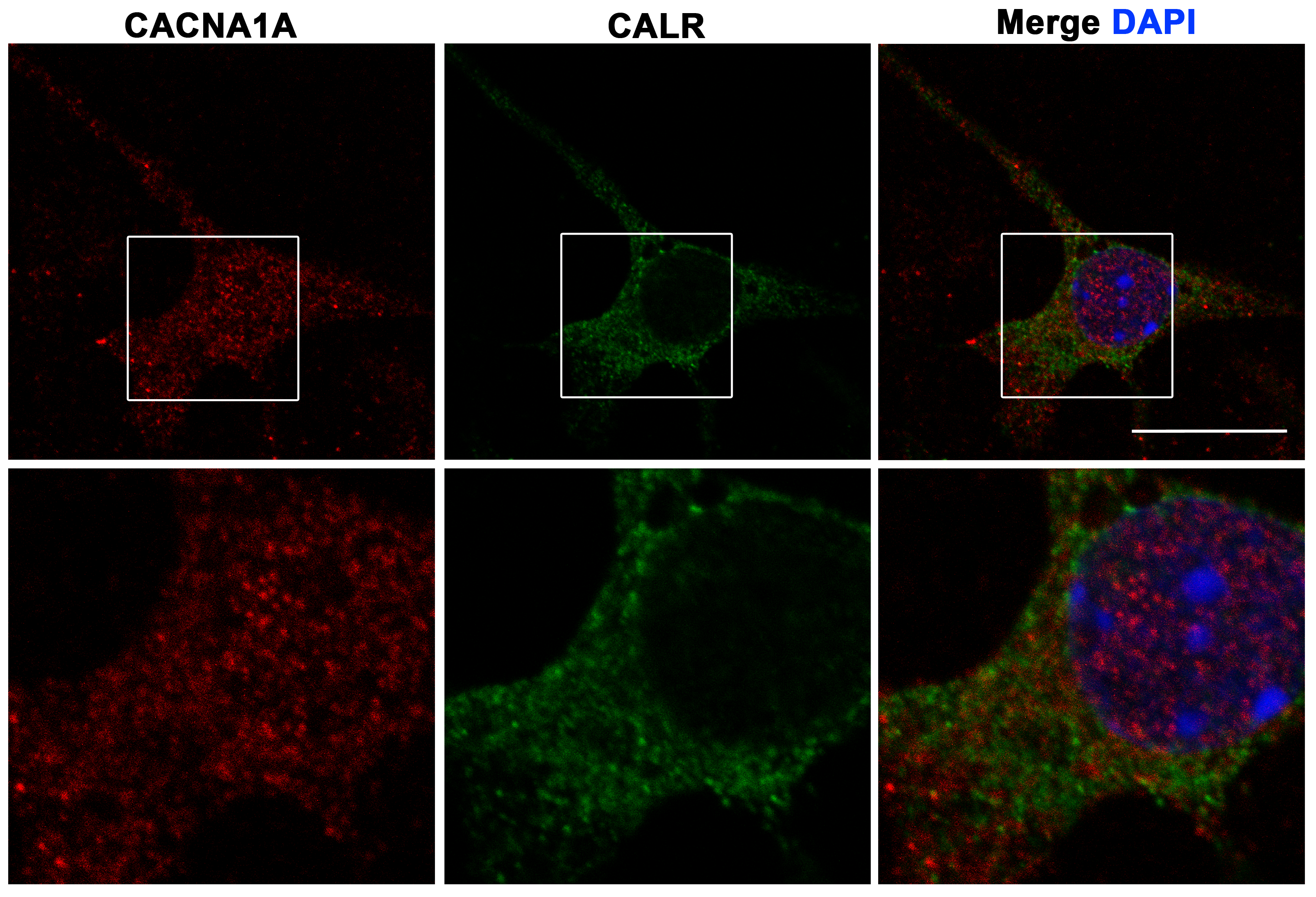

Supplement: S8 Fig — The primary cerebella neurons were stained with anti-CACNA1A antibody (red) and ER marker anti-Calreticulin (CARL, green) antibody. Very few CACNA1A punctae co-localize with CARL positive ER structures. Scale bar, 20 μm. (TIF) [file pbio.1002103.s009.tif]

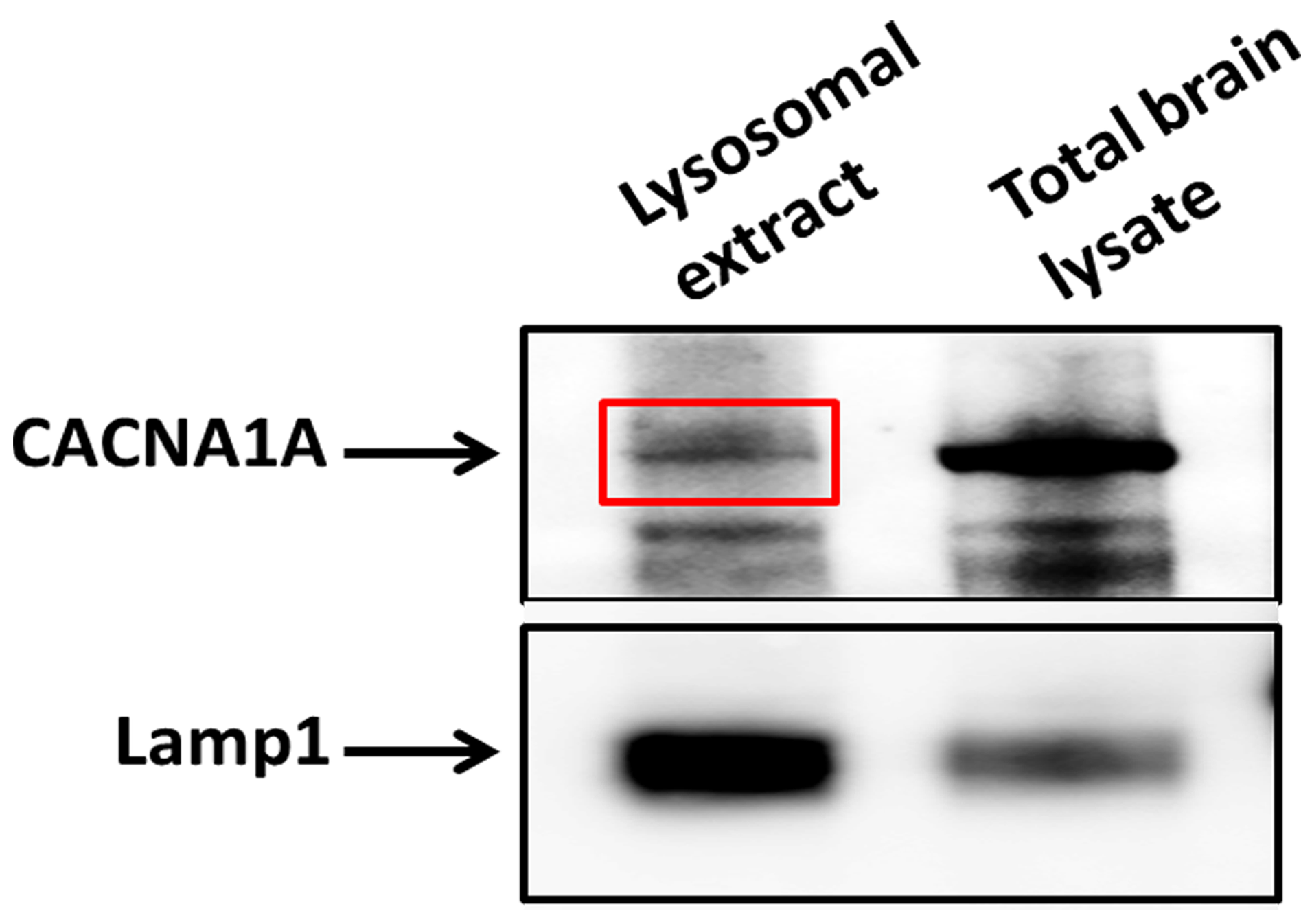

Supplement: S9 Fig — The total brain lysate and the lysosomal enriched lysates were blotted with CACNA1A and LAMP1 antibodies. A band for CACNA1A protein was detected in the lysosomal lysate. (TIF) [file pbio.1002103.s010.tif]

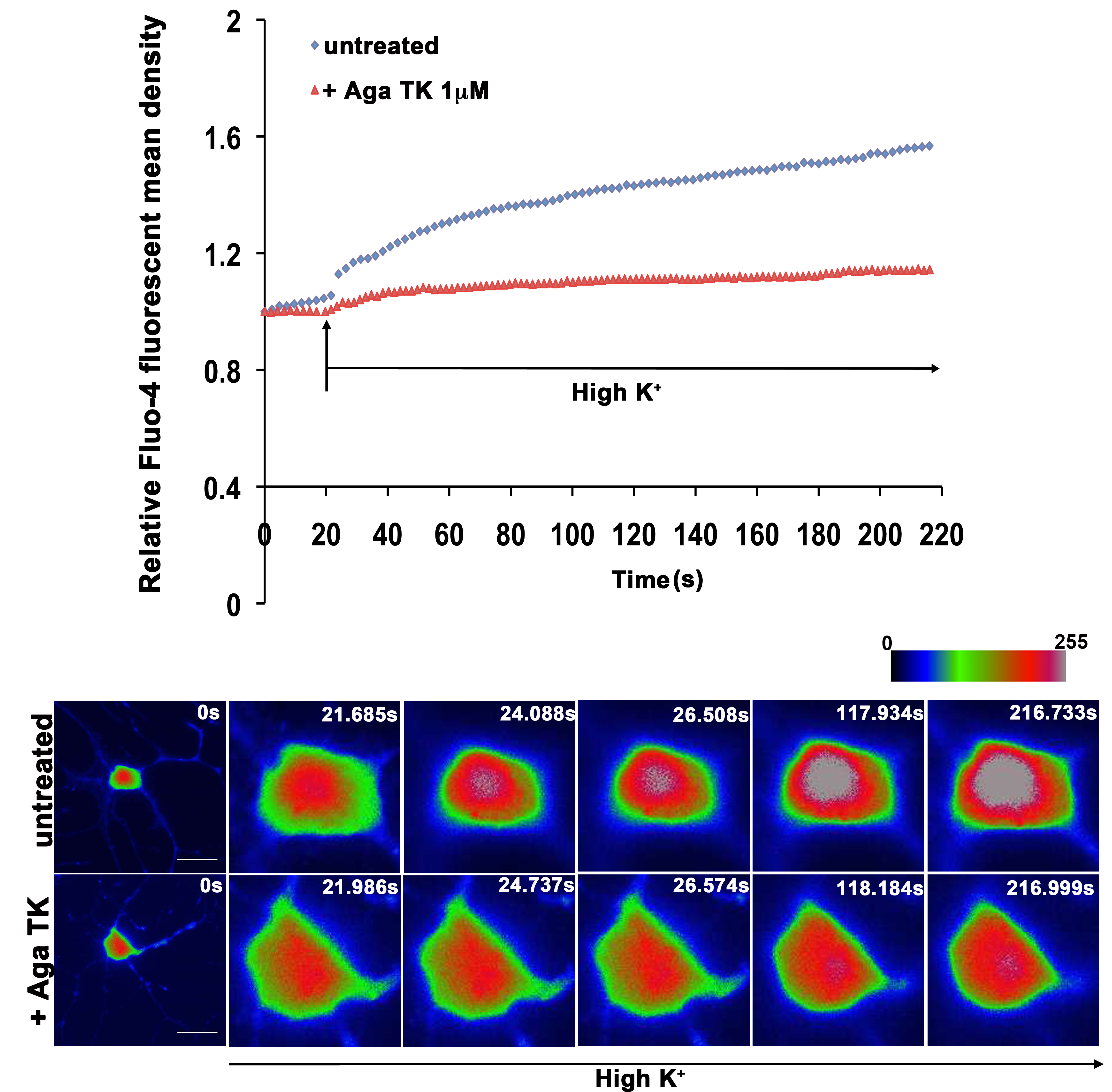

Supplement: S10 Fig — The primary cultured cerebellar neurons are preloaded with membrane permeable calcium indicator Fluo 4-AM. Depolarization with high potassium chloride solution activates VGCCs on plasma membrane. In the presence of ω-Agatoxin, the calcium influx through VGCCs is greatly reduced. (TIF) [file pbio.1002103.s011.tif]
