# Supplementary material for: A Voltage-Gated Calcium Channel Regulates Lysosomal Fusion with Endosomes and Autophagosomes and Is Required for Neuronal Homeostasis
Source: PLoS Biol. 2015 Mar 26;13(3):e1002103. doi: 10.1371/journal.pbio.1002103 (PMC4374850; doi:10.1371/journal.pbio.1002103)
Supplement: Supplementary file 3 [file pbio.1002103.s013.docx]

```
                                                                  Section 1
                 (1)  1        10        20        30               49
cac              (1)  -------------------------------------------------
CACNA1A xenopus  (1)  MARFGDEVPARGGGAG-------------------RGSRQGGPPGAQRI
CACNA1A mouse    (1)  MARFGDEMPGRYGAGGGGSGPAAGVVVGAAGGRGAGGSRQGGQPGAQRM
CACNA1A rat      (1)  MARFGDEMPGRYGAGGGGSGPAAGVVVGAAGGRGAGGSRQGGQPGAQRM
CACNA1A_i4human  (1)  MARFGDEMPARYGGGG--SGAAAGVVVGSGGGRGAGGSRQGGQPGAQRM
Consensus        (1)  MARFGDEMPARYGAGGG SG AAGVVVGAAGGRGAGGSRQGGQPGAQRM
                                                                  Section 2
                (50)  50        60        70        80               98
cac              (1)  -------------MGGPKKEENPPGGGPTSLFILTEDNPIRKYTRFIIE
CACNA1A xenopus (32)  YKQSMAQRARTMALYNPIPVRQNCLTVNRSLFLFSEDNVVRKYAKKITE
CACNA1A mouse   (50)  YKQSMAQRARTMALYNPIPVRQNCLTVNRSLFLFSEDNVVRKYAKKITE
CACNA1A rat     (50)  YKQSMAQRARTMALYNPIPVRQNCLTVNRSLFLFSEDNVVRKYAKKITE
CACNA1A_i4human (48)  YKQSMAQRARTMALYNPIPVRQNCLTVNRSLFLFSEDNVVRKYAKKITE
Consensus       (50)  YKQSMAQRARTMALYNPIPVRQNCLTVNRSLFLFSEDNVVRKYAKKITE
                                                                  Section 3
                (99)  99        110       120       130             147
cac             (37)  WPPFEYAVLLTIIANCVVLALEEHLPGGDKTVLAQKLEKTEAYFLCIFC
CACNA1A xenopus (81)  WPPFEYMILATIIANCIVLAMEQHLPVGDKTPMSERLDDTEPYFIGIFC
CACNA1A mouse   (99)  WPPFEYMILATIIANCIVLALEQHLPDDDKTPMSERLDDTEPYFIGIFC
CACNA1A rat     (99)  WPPFEYMILATIIANCIVLALEQHLPDDDKTPMSERLDDTEPYFIGIFC
CACNA1A_i4human (97)  WPPFEYMILATIIANCIVLALEQHLPDDDKTPMSERLDDTEPYFIGIFC
Consensus       (99)  WPPFEYMILATIIANCIVLALEQHLPDDDKTPMSERLDDTEPYFIGIFC
                                                                  Section 4
               (148)  148       160       170       180             196
cac             (86)  VEASLKILALGLVLHKHSYLRNIWNIMDFFVVVTGFMTQYPQIGPEVDL
CACNA1A xenopus(130)  FETGIKIIALGFAFHKGSYLRNGWNIMDFVVVLSGIVAS---TGPEFDL
CACNA1A mouse  (148)  FEAGIKIVALGFAFHKGSYLRNGWNVMDFVVVLTGILAT---VGTEFDL
CACNA1A rat    (148)  FEAGIKIVALGFAFHKGSYLRNGWNVMDFVVVLTGILAT---VGTEFDL
CACNA1A_i4human(146)  FEAGIKIIALGFAFHKGSYLRNGWNVMDFVVVLTGILAT---VGTEFDL
Consensus      (148)  FEAGIKIIALGFAFHKGSYLRNGWNVMDFVVVLTGILAT   VGTEFDL
                                                                  Section 5
               (197)  197          210       220       230          245
cac            (135)  RTLRAIRVLRPLKLVSGIPSLQVVLKSIIKAMAPLLQIGLLVLFAIVIF
CACNA1A xenopus(176)  RTLRAVRVLRPLKLVSGIPSLQVVLKSIMKAMIPLLQIGLLLFFAILIF
CACNA1A mouse  (194)  RTLRAVRVLRPLKLVSGIPSLQVVLKSIMKAMIPLLQIGLLLFFAILIF
CACNA1A rat    (194)  RTLRAVRVLRPLKLVSGIPSLQVVLKSIMKAMIPLLQIGLLLFFAILIF
CACNA1A_i4human(192)  RTLRAVRVLRPLKLVSGIPSLQVVLKSIMKAMIPLLQIGLLLFFAILIF
Consensus      (197)  RTLRAVRVLRPLKLVSGIPSLQVVLKSIMKAMIPLLQIGLLLFFAILIF
                                                                  Section 6
               (246)  246            260       270       280        294
cac            (184)  AIIGLEFYSGALHKTCYSLEDPNKLVKEGESETPCNTDNILEKATGSFV
CACNA1A xenopus(225)  AIIGLDFYIGKFHTSCYDMAGEDIR-----EEVLCGNDSSSRRCP----
CACNA1A mouse  (243)  AIIGLEFYMGKFHTTCFEEGTDDIQG---ESPAPCGTEEPARTCP----
CACNA1A rat    (243)  AIIGLEFYMGKFHTTCFEEGTDDIQG---ESPAPCGTEEPARTCP----
CACNA1A_i4human(241)  AIIGLEFYMGKFHTTCFEEGTDDIQG---ESPAPCGTEEPARTCP----
Consensus      (246)  AIIGLEFYMGKFHTTCFEEGTDDIQG   ESPAPCGTEEPARTCP
```

```
                                                                    Section 7
                   (295) 295 300                 310                 320                 330               343
                cac (233) CNNTTSMCLEKWEGPNSGITSFDNIGFAMLTVFQCITMEGWTAILYWTN
  CACNA1A xenopus (265) ---ENTQCLSKWEGPNNGITQFDNILFAILTVFQCITMEGWTDILYYSN
    CACNA1A mouse (285) ---NGTKCQPYWEGPNNGITQFDNILFAVLTVFQCITMEGWTDLLYNSN
      CACNA1A rat (285) ---NGTKCQPYWEGPNNGITQFDNILFAVLTVFQCITMEGWTDLLYNSN
  CACNA1A_i4human (283) ---NGTKCQPYWEGPNNGITQFDNILFAVLTVFQCITMEGWTDLLYNSN
        Consensus (295)    NGTKCQPYWEGPNNGITQFDNILFAVLTVFQCITMEGWTDLLYNSN

                                                                    Section 8
                   (344) 344   350                 360                 370                 380             392
                cac (282) DALGSAFNWIYFVPLIVIGSFFMLNLVLGVLSGEFSNERNRVERRMEFQ
  CACNA1A xenopus (311) DVFGSAFNWLYFIPLIIIGSFFMLNLVLGVLSGEFAKERERVENRRAFM
    CACNA1A mouse (331) DASGNTWNWLYFIPLIIIGSFFMLNLVLGVLSGEFAKERERVENRRAFL
      CACNA1A rat (331) DASGNTWNWLYFIPLIIIGSFFMLNLVLGVLSGEFAKERERVENRRAFL
  CACNA1A_i4human (329) DASGNTWNWLYFIPLIIIGSFFMLNLVLGVLSGEFAKERERVENRRAFL
        Consensus (344) DASGNTWNWLYFIPLIIIGSFFMLNLVLGVLSGEFAKERERVENRRAFL

                                                                    Section 9
                   (393) 393    400                 410                 420                 430           441
                cac (331) KCRFRAMFQTAMVSYLDWITQAEEVILAEERTTEEEKMHIMEARRRNAA
  CACNA1A xenopus (360) KLRRQQQIERELNGYMEWIS-KAEVILAEDDSDIEQRLPYDGALRRPTI
    CACNA1A mouse (380) KLRRQQQIERELNGYMEWISKAEEVILAEDETDVEQRHPFDGALRRATL
      CACNA1A rat (380) KLRRQQQIERELNGYMEWISKAEEVILAEDETDVEQRHPFDGALRRATL
  CACNA1A_i4human (378) KLRRQQQIERELNGYMEWISKAEEVILAEDETDGEQRHPFD-ALRRTTI
        Consensus (393) KLRRQQQIERELNGYMEWISKAEEVILAEDETDVEQRHPFDGALRR TI

                                                                    Section 10
                   (442) 442     450                 460                 470                 480           490
                cac (380) KRKKLKSLGKSKSTDTEEEEAEEDYGDDGYLKTRSKPQGSCTGFWRAEK
  CACNA1A xenopus (408) KKNKTELLNAEEGEEAIGDIASVGSPFARASIKSAKME-NSSYMHKKER
    CACNA1A mouse (429) KKSKTDLLNPEEAEDQLADIASVGSPFARASIKSAKLE-NSTFFHKKER
      CACNA1A rat (429) KKSKTDLLNPEEAEDQLADIASVGSPFARASIKSAKLE-NSTFFHKKER
  CACNA1A_i4human (426) KKSKTDLLNPEEAEDQLADIASVGSPFARASIKSAKLE-NSTFFHKKER
        Consensus (442) KKSKTDLLNPEEAEDQLADIASVGSPFARASIKSAKLE NSTFFHKKER

                                                                    Section 11
                   (491) 491      500                 510                 520                           539
                cac (429) RFRFWIRHTVKTQWFYWFVIVLVFLNTVCVAVEHYGQPSFLTEFLYYAE
  CACNA1A xenopus (456) RIRFLIRRMVKTQAFYWVVLGLVALNTLCVAIVHYGQDDRLSEFLYYAE
    CACNA1A mouse (477) RMRFYIRRMVKTQAFYWTVLSLVALNTLCVAIVHYNQPEWLSDFLYYAE
      CACNA1A rat (477) RMRFYIRRMVKTQAFYWTVLSLVALNTLWLAIVHYNQPEWLSDFLYYAE
  CACNA1A_i4human (474) RMRFYIRRMVKTQAFYWTVLSLVALNTLCVAIVHYNQPEWLSDFLYYAE
        Consensus (491) RMRFYIRRMVKTQAFYWTVLSLVALNTLCVAIVHYNQPEWLSDFLYYAE

                                                                    Section 12
                   (540) 540       550                 560                 570                           588
                cac (478) FIFLGLFMSEMFIKMYALGPRIYFESSFNRFDCVVISGSIFEVIWSEVK
  CACNA1A xenopus (505) FIFLGLFMSEMFIKMYGLGTRPYFHSSFNCFDCAVITGSIFEVVWAMVK
    CACNA1A mouse (526) FIFLGLFMSEMFIKMYGLGTRPYFHSSFNCFDCGVIIGSIFEVIWAVIK
      CACNA1A rat (526) FIFLGLFMSEMFIKMYGLGTRPYFHSSFNCFDCGVIIGSIFEVIWAVIK
  CACNA1A_i4human (523) FIFLGLFMSEMFIKMYGLGTRPYFHSSFNCFDCGVIIGSIFEVIWAVIK
        Consensus (540) FIFLGLFMSEMFIKMYGLGTRPYFHSSFNCFDCGVIIGSIFEVIWAVIK
```

```
                                                                    Section 13
                         (589) 589        600       610       620             637
                  cac    (527) -GGSFGLSVLRALRLLRIFKVTKYWSSLRNLVISLLNSMRSIISLLFLL
       CACNA1A xenopus   (554) PGTSFGISVLRALRLLRIFKVTKYWASLRNLVISLLSSMKSIISLLFLL
         CACNA1A mouse   (575) PGTSFGISVLRALRLLRIFKVTKYWASLRNLVVSLLNSMKSIISLLFLL
           CACNA1A rat   (575) PGTSFGISVLRALRLLRIFKVTKYWASLRNLVVSLLNSMKSIISLLFLL
      CACNA1A_i4human    (572) PGTSFGISVLRALRLLRIFKVTKYWASLRNLVVSLLNSMKSIISLLFLL
            Consensus    (589) PGTSFGISVLRALRLLRIFKVTKYWASLRNLVVSLLNSMKSIISLLFLL
                                                                    Section 14
                         (638) 638         650       660       670            686
                  cac    (575) FLFILIFALLGMQLFGGQFNLPGGTPETNFNTFPIALLTVFQILTGEDW
       CACNA1A xenopus   (603) FLFIVVFALLGMQLFGGQFNFDEKTPATNFDTFPAAIMTVFQILTGEDW
         CACNA1A mouse   (624) FLFIVVFALLGMQLFGGQFNFDEGTPPTNFDTFPAAIMTVFQILTGEDW
           CACNA1A rat   (624) FLFIVVFALLGMQLFGGQFNFDEGTPPTNFDTFPAAIMTVFQILTGEDW
      CACNA1A_i4human    (621) FLFIVVFALLGMQLFGGQFNFDEGTPPTNFDTFPAAIMTVFQILTGEDW
            Consensus    (638) FLFIVVFALLGMQLFGGQFNFDEGTPPTNFDTFPAAIMTVFQILTGEDW
                                                                    Section 15
                         (687) 687          700       710       720           735
                  cac    (624) NEVMYQGIISQGGAQKGMIYSIYFIVLVLFGNYTLLNVFLAIAVDNLAN
       CACNA1A xenopus   (652) NAVMYDGIKSQGGVKTGMVFSIYFIILTLFGNYTLLNVFLAIAVDNLAN
         CACNA1A mouse   (673) NEVMYDGIKSQGGVQGGMVFSIYFIVLTLFGNYTLLNVFLAIAVDNLAN
           CACNA1A rat   (673) NEVMYDEIKSQGGVQGGMVFSIYFIVLTLFGNYTLLNVFLAIAVDNLAN
      CACNA1A_i4human    (670) NEVMYDGIKSQGGVQGGMVFSIYFIVLTLFGNYTLLNVFLAIAVDNLAN
            Consensus    (687) NEVMYDGIKSQGGVQGGMVFSIYFIVLTLFGNYTLLNVFLAIAVDNLAN
                                                                    Section 16
                         (736) 736           750       760       770         784
                  cac    (673) AQELTAAEEEQVEEDKEKQLQELEKEMEALQADGVHVENGDGAVAPSK-
       CACNA1A xenopus   (701) AQELTKDEQE--EEEAANQKLAMQKAKEVAEVSPLSATNMSIAAKEQQK
         CACNA1A mouse   (722) AQELTKDEQE--EEEAANQKLALQKAKEVAEVSPLSAANMSIAVKEQQK
           CACNA1A rat   (722) AQELTKDEQE--EEEAANQKLALQKAKEVAEVSPLSAANMSIAVKEQQK
      CACNA1A_i4human    (719) AQELTKDEQE--EEEAANQKLALQKAKEVAEVSPLSAANMSIAVKEQQK
            Consensus    (736) AQELTKDEQE  EEEAANQKLALQKAKEVAEVSPLSAANMSIAVKEQQK
                                                                    Section 17
                         (785) 785  790       800       810       820           833
                  cac    (721) ----------------------SK-------------------------
       CACNA1A xenopus   (748) NQKTSMSVWEQRTNEIRRQNLMNSREALYSELDPEERWKLSFMR---PD
         CACNA1A mouse   (769) NQKPTKSVWEQRTSEMRKQNLLASREALY--GDAAERWPTPYARPLRPD
           CACNA1A rat   (769) NQKPAKSVWEQRTSEMRKQNLLASREALY--GDAAERWPTTYARPLRPD
      CACNA1A_i4human    (766) NQKPAKSVWEQRTSEMRKQNLLASREALYNEMDPDERWKAAYTRHLRPD
            Consensus    (785) NQKPAKSVWEQRTSEMRKQNLLASREALY   D  ERW   Y R LRPD
                                                                    Section 18
                         (834) 834   840       850       860       870          882
                  cac    (723) -------------------------------------------------
       CACNA1A xenopus   (794) VKTHLDRPLVVDPQENRNNNTNKTRPGEGRNPIEQRLSQQRAEDYLRKQ
         CACNA1A mouse   (816) VKTHLDRPLVVDPQENRNNNTNKS----------------RAPEALRPT
           CACNA1A rat   (816) VKTHLDRPLVVDPQENRNNNTNKS----------------RAPEALRQT
      CACNA1A_i4human    (815) MKTHLDRPLVVDPQENRNNNTNKSRAAEPT--VDQRLGQQRAEDFLRKQ
            Consensus    (834) VKTHLDRPLVVDPQENRNNNTNKS                RA D LR
```

```
                                                        Section 19
                  (883) 883    890       900       910       920      931
              cac (723) -------------------------------------------------
  CACNA1A xenopus (843) ARFQERGGRG--------YDMG-----QEPGRR-PRIKEQDIIRDGQDL
    CACNA1A mouse (849) ARPRESARDP------DARRAWPGSPERAPGREGPYGRESEPQQRDHAP
      CACNA1A rat (849) ARPRESARDP------DARRAWPSSPERAPGREGPYGRESEPQQREHAP
CACNA1A_i4human (862) ARYHDRARDPSGSAGLDARRPWAGSQEAELSREGPYGRESDHHAREGS-
        Consensus (883) AR  E ARDP      DARR W  S E  PGREGPYGRESD   RE A

                                                        Section 20
                  (932) 932     940       950       960       970     980
              cac (723) ------------------------------------GK-----------
  CACNA1A xenopus (878) GMQDRTGEMDGGIRPPQDPHRRHRGGGSKE--SRSGSPLPRGDGDHRRH
    CACNA1A mouse (892) PREHAPWDADTERAKAGDAPRRHTHRPVAE-------------GEPRRH
      CACNA1A rat (892) PREHVPWDADPERAKAGDAPRRHTHRPVAE-------------GEPRRH
CACNA1A_i4human (910) LEQPGFWEGEAERGKAGDPHRRHVHRQGGSRESRSGSPRTGADGEHRRH
        Consensus (932)       WDAD ERAKAGD  RRH HR  AE             GE RRH

                                                        Section 21
                  (981) 981      990       1000      1010              1029
              cac (725) -------------------------------------------------
  CACNA1A xenopus (925) RPHRRTGEDNEDAGGGGGGGGRGGGGGGDRRPRHGRDGMRVREGGGGES
    CACNA1A mouse (928) RARRRPG-DEPDDRP-------------ERRPRP-RDATRPARAADGEG
      CACNA1A rat (928) RARRRPG-DEPDDRP-------------ERRPRP-RDATRPARAADGEG
CACNA1A_i4human (959) RAHRRPGEEGPEDKA-------------ERRARH-REGSRPARGGEGEG
        Consensus (981) RA RRPG D PDDR              ERRPR  RDATRPARAADGEG

                                                        Section 22
                 (1030) 1030     1040      1050      1060             1078
              cac (725) -------------------------------------------------
  CACNA1A xenopus (974) EG-PEGERRRRHRHAAQSTYESDAKRDDRERRHRRRKEPTAPTPGSGGP
    CACNA1A mouse (962) ---DDGERKRRHRHGPPAHDDR-------ERRHRRRKENQGSGVPVSGP
      CACNA1A rat (962) ---DDGERKRRHRHGPPAHDDR-------ERRHRRRKESQGSGVPMSGP
CACNA1A_i4human (994) EGPDGGERRRRHRHGAPATYEGDARREDKERRHRRRKENQGSGVPVSGP
       Consensus (1030)    DDGERKRRHRHG PA  D        ERRHRRRKE QGSGVPVSGP

                                                        Section 23
                 (1079) 1079      1090      1100      1110            1127
              cac (725) -------------------------------------------------
 CACNA1A xenopus (1022) NLSTTRPIQDRSRQENQNQQPENMDNIRNTKLVTSE---PPAGGGVGGL
   CACNA1A mouse (1001) NLSTTRPIQQDLGRQDLP-LAEDLDNMKNNKLATGEPASPHDSLGHSGL
     CACNA1A rat (1001) NLSTTRPIQQDLGRQDLP-LAEDLDNMKNNKLATGEPASPHDSLGHSGL
CACNA1A_i4human (1043) NLSTTRPIQQDLGRQDPP-LAEDIDNMKNNKLATAESAAPHGSLGHAGL
       Consensus (1079) NLSTTRPIQQDLGRQD P LAEDLDNMKNNKLATGE ASPH SLGHSGL

                                                        Section 24
                 (1128) 1128       1140      1150      1160           1176
              cac (725) -------------------------------------------------
 CACNA1A xenopus (1068) NHSPAKIGNHTNCAN---------NNSVPTGNATPRRTPHPTNSGPPPS
   CACNA1A mouse (1049) PPSPAKIGNSTNPG-----PALATNPQNAASRRTPNNPGNPSNPGPPKT
     CACNA1A rat (1049) PPSPAKIGNSTNPG-----PALATNPQNAASRRTPNNPGNPSNPGPPKT
CACNA1A_i4human (1091) PQSPAKMGNSTDPGPMLAIPAMATNPQNAASRRTPNNPGNPSNPGPPKT
       Consensus (1128) P SPAKIGNSTNPG     PALATNPQNAASRRTPNNPGNPSNPGPPKT
```

```
                                                                              Section 25
                     (1177) 1177         1190      1200      1210           1225
               cac    (725) ----------------------------------KKE------------
CACNA1A xenopus      (1108) ---APDPSHTFSNPTETAKGTKTTEHTTVDIPPPFPPPVENAIGQANKI
CACNA1A mouse        (1093) PENSLIVTNPSSTQPNSAKTARKPEHMAVEIPPACPP-LNHTVVQVNKN
CACNA1A rat          (1093) PENSLIVTNPSSTQPNSAKTARKPEHMAVEIPPACPP-LNHTVVQVNKN
CACNA1A_i4human      (1140) PENSLIVTNPSGTQTNSAKTARKPDHTTVDIPPACPPPLNHTVVQVNKN
Consensus            (1177) PENSLIVTNPSSTQ NSAKTARKPEH  VDIPPACPP LNHTVVQVNKN
                                                                              Section 26
                     (1226) 1226          1240      1250      1260          1274
               cac    (728) -----E-----EKKEEE---E-VTEGPKPMLPYSSMFILSPTNPIRRGA
CACNA1A xenopus      (1154) VAVSPLPKKDDEKKEEEEDDDDGEKGPKPMPPYSSMFILSTTNPLRRLC
CACNA1A mouse        (1141) ANPDPLPKKEEEKKEEE-EADPGEDGPKPMPPYSSMFILSTTNPLRRLC
CACNA1A rat          (1141) ANPDPLPKKEEEKKEEE-EADPGEDGPKPMPPYSSMFILSTTNPLRRLC
CACNA1A_i4human      (1189) ANPDPLPKKEEEKKEEE-EDDRGEDGPKPMPPYSSMFILSTTNPLRRLC
Consensus            (1226) ANPDPLPKKEEEKKEEE E D GEDGPKPMPPYSSMFILSTTNPLRRLC
                                                                              Section 27
                     (1275) 1275 1280      1290      1300      1310         1323
               cac    (763) HWVVNLPYFDFFIMVVISMSSIALAAEDPVRENSRRNKILNYFDYAFTG
CACNA1A xenopus      (1203) HYIVTMRYFEMCILMVIAMSSIALAAEDPVQPDAPRNNVLRYFDYVFTG
CACNA1A mouse        (1189) HYILNLRYFEMCILMVIAMSSIALAAEDPVQPNAPRNNVLRYFDYVFTG
CACNA1A rat          (1189) HYILNLRYFEMCILMVIAMSSIALAAEDPVQPNAPRNNVLRYFDYVFTG
CACNA1A_i4human      (1237) HYILNLRYFEMCILMVIAMSSIALAAEDPVQPNAPRNNVLRYFDYVFTG
Consensus            (1275) HYILNLRYFEMCILMVIAMSSIALAAEDPVQPNAPRNNVLRYFDYVFTG
                                                                              Section 28
                     (1324) 1324  1330      1340      1350      1360         1372
               cac    (812) VFTIEMLLKIVDLGVILHPGSYLREFWNIMDAVVVICAAVSFGFDMS--
CACNA1A xenopus      (1252) VFTFEMVIKMVDLGLILHQGAYFRDLWNFLDFIVVCGALVAFAFSSNQR
CACNA1A mouse        (1238) VFTFEMVIKMIDLGLVLHQGAYFRDLWNILDFIVVSGALVAFAFTGN--
CACNA1A rat          (1238) VFTFEMVIKMIDLGLVLHQGAYFRDLWNILDFIVVSGALVAFAFTGN--
CACNA1A_i4human      (1286) VFTFEMVIKMIDLGLVLHQGAYFRDLWNILDFIVVSGALVAFAFTGN--
Consensus            (1324) VFTFEMVIKMIDLGLVLHQGAYFRDLWNILDFIVVSGALVAFAFTGN
                                                                              Section 29
                     (1373) 1373   1380      1390      1400      1410         1421
               cac    (859) GSSAGQNLSTIKSLRVLRVLRPLKTIKRVPKLKAVFDCVVNSLKNVVNI
CACNA1A xenopus      (1301) GGSKGKDISTIKSLRVLRVLRPLKTIKRLPKLKAVFDCVVNSLKNVLNI
CACNA1A mouse        (1285) --SKGKDINTIKSLRVLRVLRPLKTIKRLPKLKAVFDCVVNSLKNVFNI
CACNA1A rat          (1285) --SKGKDINTIKSLRVLRVLRPLKTIKRLPKLKAVFDCVVNSLKNVFNI
CACNA1A_i4human      (1333) --SKGKDINTIKSLRVLRVLRPLKTIKRLPKLKAVFDCVVNSLKNVFNI
Consensus            (1373)   SKGKDINTIKSLRVLRVLRPLKTIKRLPKLKAVFDCVVNSLKNVFNI
                                                                              Section 30
                     (1422) 1422    1430      1440      1450      1460       1470
               cac    (908) LIVYILFQFIFSVIGVQLFNGKFFYCTDESKHTSAECQGSYFKYEEDEL
CACNA1A xenopus      (1350) LIVYMLFMFIFAVVAVQLFKGKFFYCTDESKEFQRDCRGEYLVYDKSEV
CACNA1A mouse        (1332) LIVYMLFMFIFAVVAVQLFKGKFFHCTDESKEFERDCRGKYLLYEKNEV
CACNA1A rat          (1332) LIVYMLFMFIFAVVAVQLFKGKFFHCTDESKEFERDCRGKYLLYEKNEV
CACNA1A_i4human      (1380) LIVYMLFMFIFAVVAVQLFKGKFFHCTDESKEFEKDCRGKYLLYEKNEV
Consensus            (1422) LIVYMLFMFIFAVVAVQLFKGKFFHCTDESKEFERDCRGKYLLYEKNEV
```

```
                                                                                      Section 31
                  (1471) 1471     1480      1490      1500                   1519
                cac (957) LPKQELRVWKPRAFHYDNVAAAMLTLFAVQTGEGWPQVLQHSMAATYED
   CACNA1A xenopus (1399) KAQK--REWKKWPFHYDNVLWALLTLFTVSTGEGWPDVLRHSVDATYED
     CACNA1A mouse (1381) KARD--REWKKYEFHYDNVLWALLTLFTVSTGEGWPQVLKHSVDATFEN
       CACNA1A rat (1381) KARD--REWKKYDFHYDNVLWALLTLFTVSTGEGWPQVLKHSVDATFEN
   CACNA1A_i4human (1429) KARD--REWKKYEFHYDNVLWALLTLFTVSTGEGWPQVLKHSVDATFEN
         Consensus (1471) KARD  REWKKYEFHYDNVLWALLTLFTVSTGEGWPQVLKHSVDATFEN
                                                                                      Section 32
                  (1520) 1520      1530      1540      1550                 1568
               cac (1006) RGPIQNFRIEMSIFYIVYFIVFPFFFVNIFVALIIITFQEQGEAELQDG
   CACNA1A xenopus (1446) QGPRPGFRMEMSIFYVVYFVVFPFFFVNIFVALIIITFQEQGDKIMEEY
     CACNA1A mouse (1428) QGPSPGYRMEMSIFYVVYFVVFPFFFVNIFVALIIITFQEQGDKMMEEY
       CACNA1A rat (1428) QGPSPGYRMEMSIFYVVYFVVFPFFFVNIFVALIIITFQEQGDKMMEEY
   CACNA1A_i4human (1476) QGPSPGYRMEMSIFYVVYFVVFPFFFVNIFVALIIITFQEQGDKMMEEY
         Consensus (1520) QGPSPGYRMEMSIFYVVYFVVFPFFFVNIFVALIIITFQEQGDKMMEEY
                                                                                      Section 33
                  (1569) 1569       1580      1590      1600                1617
               cac (1055) EIDKNQKSCIDFTIGARPLERYMPKNRNTFKYKVWRIVVSTPFEYFIMM
   CACNA1A xenopus (1495) SLEKNERACIDFAISAKPLTRHMPQNKQSFQYRMWQFVVSPPFEYTIMA
     CACNA1A mouse (1477) SLEKNERACIDFAISAKPLTRHMPQNKQSFQYRMWQFVVSPPFEYTIMA
       CACNA1A rat (1477) SLEKNERACIDFAISAKPLTRHMPQNKQSFQYRMWQFVVSPPFEYTIMA
   CACNA1A_i4human (1525) SLEKNERACIDFAISAKPLTRHMPQNKQSFQYRMWQFVVSPPFEYTIMA
         Consensus (1569) SLEKNERACIDFAISAKPLTRHMPQNKQSFQYRMWQFVVSPPFEYTIMA
                                                                                      Section 34
                  (1618) 1618        1630      1640      1650               1666
               cac (1104) LIVFNTLLLMMKYHNQGDMYEKSLKYINMGFTGMFSVETVLKIIGFGVK
   CACNA1A xenopus (1544) LIALNTIVLMMKFHGSSNTYENVLKTFNMVFTSLFSLECLLKIMAFGAP
     CACNA1A mouse (1526) MIALNTIVLMMKFYGASVAYENALRVFNIVFTSLFSLECVLKVMAFGIL
       CACNA1A rat (1526) MIALNTIVLMMKFYGASVAYENALRVFNIVFTSLFSLECVLKVMAFGIL
   CACNA1A_i4human (1574) MIALNTIVLMMKFYGASVAYENALRVFNIVFTSLFSLECVLKVMAFGIL
         Consensus (1618) MIALNTIVLMMKFYGASVAYENALRVFNIVFTSLFSLECVLKVMAFGIL
                                                                                      Section 35
                  (1667) 1667         1680      1690      1700              1715
               cac (1153) NFFKDPWNIFDLITVLGSIVDALWMEFGHDSNSINVGFLRLFRAARLIK
   CACNA1A xenopus (1593) NYFRDAWNIFDFVTVLGSITDIVVTELWDS--IINLSFLRLFRAARLIK
     CACNA1A mouse (1575) NYFRDAWNIFDFVTVLGSITDILVTEFGNN--FINLSFLRLFRAARLIK
       CACNA1A rat (1575) NYFRDAWNIFDFVTVLGSITDILVTEFGNN--FINLSFLRLFRAARLIK
   CACNA1A_i4human (1623) NYFRDAWNIFDFVTVLGSITDILVTEFGNN--FINLSFLRLFRAARLIK
         Consensus (1667) NYFRDAWNIFDFVTVLGSITDILVTEFGNN  FINLSFLRLFRAARLIK
                                                                                      Section 36
                  (1716) 1716          1730      1740      1750             1764
               cac (1202) LLRQGYTIRILLWTFVQSFKALPYVCLLIAMLFFIYAIIGMQVFGNIKL
   CACNA1A xenopus (1640) LLRQGYTIRILLWTFVQSFKALPYVCLLIAMLFFIYAIIGMQVFGNIAL
     CACNA1A mouse (1622) LLRQGYTIRILLWTFVQSFKALPYVCLLIAMLFFIYAIIGMQVFGNIGI
       CACNA1A rat (1622) LLRQGYTIRILLWTFVQSFKALPYVCLLIAMLFFIYAIIGMQVFGNIGI
   CACNA1A_i4human (1670) LLRQGYTIRILLWTFVQSFKALPYVCLLIAMLFFIYAIIGMQVFGNIGI
         Consensus (1716) LLRQGYTIRILLWTFVQSFKALPYVCLLIAMLFFIYAIIGMQVFGNIGI
```

```
                                                                         Section 37
             (1765) 1765 1770      1780      1790      1800          1813
           cac (1251) GTVENS-------ITRHNNFQSFIQGVMLLFRCATGEAWPNIMLACLKG
CACNA1A xenopus (1689) DEEDDD-----TAITEHNNFRNFFQALMLLFRSATGEAWHEIMLSCLSG
  CACNA1A mouse (1671) DGEDEDSDEDEFQITEHNNFRTFFQALMLLFRSATGEAWHNIMLSCLSG
    CACNA1A rat (1671) DGEDEDSDEDEFQITEHNNFRTFFQALMLLFRSATGEAWHNIMLSCLSG
CACNA1A_i4human (1719) DVEDEDSDEDEFQITEHNNFRTFFQALMLLFRSATGEAWHNIMLSCLSG
      Consensus (1765) D EDEDSDEDEFQITEHNNFRTFFQALMLLFRSATGEAWHNIMLSCLSG
                                                                         Section 38
             (1814) 1814  1820      1830      1840      1850         1862
           cac (1293) KACDDDAEKAPGEYCGSTLAYAYFVSFIFFCSFLMLNLFVAVIMDNFDY
CACNA1A xenopus (1733) RPCDVGSGLKE-KECGSEFAYFYFVSFIFLCSFLMLNLFVAVIMDNFEY
  CACNA1A mouse (1720) KPCDKNSGILT-ADCGNEFAYFYFVSFIFLCSFLMLNLFVAVIMDNFEY
    CACNA1A rat (1720) KPCDKNSGIQK-PECGNEFAYFYFVSFIFLCSFLMLNLFVAVIMDNFEY
CACNA1A_i4human (1768) KPCDKNSGILT-RECGNEFAYFYFVSFIFLCSFLMLNLFVAVIMDNFEY
      Consensus (1814) KPCDKNSGI    ECGNEFAYFYFVSFIFLCSFLMLNLFVAVIMDNFEY
                                                                         Section 39
             (1863) 1863    1870      1880      1890      1900       1911
           cac (1342) LTRDSSILGAHHLDEFVRIWAEYDPNATGKIHYTEMYDMLKNMDPPLGF
CACNA1A xenopus (1781) LTRDSSILGPHHLDEYVRVWAEYDPAACGRIHYKDMYSLLRVISPPLGL
  CACNA1A mouse (1768) LTRDSSILGPHHLDEYVRVWAEYDPAACGRIHYKDMYSLLRVISPPLGL
    CACNA1A rat (1768) LTRDSSILGPHHLDEYVRVWAEYDPAACGRIHYKDMYSLLRVISPPLGL
CACNA1A_i4human (1816) LTRDSSILGPHHLDEYVRVWAEYDPAAWGRMPYLDMYQMLRHMSPPLGL
      Consensus (1863) LTRDSSILGPHHLDEYVRVWAEYDPAACGRIHYKDMYSLLRVISPPLGL
                                                                         Section 40
             (1912) 1912     1920      1930      1940      1950       1960
           cac (1391) GNKCPNRLAYKKLIRMNMPLDDELRVQFTTTLFALIRENLSIKMRAP-E
CACNA1A xenopus (1830) GKKCPHRVACKRLLRMDLPVADDNTVHFNSTLMALIRTALDIKIAKGGA
  CACNA1A mouse (1817) GKKCPHRVACKRLLRMDLPVADDNTVHFNSTLMALIRTALDIKIAKGGA
    CACNA1A rat (1817) GKKCPHRVACKRLLRMDLPVADDNTVHFNSTLMALIRTALDIKIAKGGA
CACNA1A_i4human (1865) GKKCPARVAYKRLLRMDLPVADDNTVHFNSTLMALIRTALDIKIAKGGA
      Consensus (1912) GKKCPHRVACKRLLRMDLPVADDNTVHFNSTLMALIRTALDIKIAKGGA
                                                                         Section 41
             (1961) 1961     1970      1980      1990                 2009
           cac (1439) EMDQADMELRETITNIWPLQAKKMLNLLVPPSDQLNKGKLSVGKIYAGF
CACNA1A xenopus (1879) DKQQMDSQLRKEMMAIWPNMSQKTLDLLVTPHKSTD---LTVGKIYAAM
  CACNA1A mouse (1866) DKQQMDAELRKEMMAIWPNLSQKTLDLLVTPHKSTD---LTVGKIYAAM
    CACNA1A rat (1866) DKQQMDAELRKEMMAIWPNLSQKTLDLLVTPHKSTD---LTVGKIYAAM
CACNA1A_i4human (1914) DKQQMDAELRKEMMAIWPNLSQKTLDLLVTPHKSTD---LTVGKIYAAM
      Consensus (1961) DKQQMDAELRKEMMAIWPNLSQKTLDLLVTPHKSTD   LTVGKIYAAM
                                                                         Section 42
             (2010) 2010      2020      2030      2040               2058
           cac (1488) LILESWRSTRFGQLDSGMPKQSFFNCLLDMAALDKGGSRQGSISFEPNG
CACNA1A xenopus (1925) MIMEYYRQSKAKRLQALQEEQNRTPLMFQRMEPPSPTQDGPLGINALPS
  CACNA1A mouse (1912) MIMEYYRQSKAKKLQAMREEQNRTPLMFQRMEPPSPTQEGGPSQNALPS
    CACNA1A rat (1912) MIMEYYRQSKAKKLQAMREEQNRTPLMFQRMEPPSPTQEGGPSQNALPS
CACNA1A_i4human (1960) MIMEYYRQSKAKKLQAMREEQDRTPLMFQRMEPPSPTQEGGPGQNALPS
      Consensus (2010) MIMEYYRQSKAKKLQAMREEQNRTPLMFQRMEPPSPTQEGGP QNALPS
```

Section 43

```
                 (2059) 2059       2070      2080      2090          2107
               cac (1537) EGAANSQTHLLASTHHHHANGDAEHNSLATLARRSTIRKRSVRNKKMLE
  CACNA1A xenopus (1974) SQAGEMG--LP-EGGMKESQSWMTQRAQQMFQKTGTWSPERARTQENTN
    CACNA1A mouse (1961) TQLDPGGGLMAHEGGMKESPSWVTQRAQEMFQKTGTWSPERGPPIDMPN
      CACNA1A rat (1961) TQLDPGGGLMAQESSMKESPSWVTQRAQEMFQKTGTWSPERGPPIDMPN
 CACNA1A_i4human (2009) TQLDPGGALMAHESGLKESPSWVTQRAQEMFQKTGTWSPEQGPPTDMPN
        Consensus (2059) TQLDPGGGLMA ESGMKESPSWVTQRAQEMFQKTGTWSPERGPP DMPN
```

Section 44

```
                 (2108) 2108        2120      2130      2140         2156
               cac (1586) LQDASRHPSQESLTGADAGHLHPGHSYMNGHRRSPSLRHNGSPLARSPS
  CACNA1A xenopus (2020) SWPNSQQSVEMSDMGRDGYSDSEQYLVMEGHGRAASMPRLPADNQQRRK
    CACNA1A mouse (2010) SQPNSQ-SVEMREMGTDGYSDSEHYLPMEGQTRAASMPRLPAENQ-RRR
      CACNA1A rat (2010) SQPNSQ-SVEMREMGTDGYSDSEHYLPMEGQTRAASMPRLPAENQ-RRR
 CACNA1A_i4human (2058) SQPNSQ-SVEMREMGRDGYSDSEHYLPMEGQGRAASMPRLPAENQ-RRR
        Consensus (2108) SQPNSQ SVEMREMG DGYSDSEHYLPMEGQ RAASMPRLPAENQ RRR
```

Section 45

```
                 (2157) 2157         2170      2180      2190         2205
               cac (1635) PRRRG--HQYIHHDIGFSDTVSNVVEMVKETRHPRHGNSHPRYPRGSWS
  CACNA1A xenopus (2069) TRTRGSNLSTITDGSPMKRSASMLGHSQSKGIRLDEYSLERVTPEEGPR
    CACNA1A mouse (2057) GRPRGNDLSTISDTSPMKRSASVLGP---KARRLDDYSLERVPPEEN-Q
      CACNA1A rat (2057) GRPRGNNLSTISDTSPMKRSASVLGP---KARRLDDYSLERVPPEEN-Q
 CACNA1A_i4human (2105) GRPRGNNLSTISDTSPMKRSASVLGP---KARRLDDYSLERVPPEEN-Q
        Consensus (2157) GRPRGNNLSTISDTSPMKRSASVLGP   KARRLDDYSLERVPPEEN Q
```

Section 46

```
                 (2206) 2206          2220      2230      2240       2254
               cac (1682) ASTSPARSPSPSRYGGHLSRSKRTQLPYPTYGTTSLCQRSRSPSPARLQ
  CACNA1A xenopus (2118) HGHQRRRERGHRVSDRSLTRYVDLESGLGTELSTTTQSCDLPTE----E
    CACNA1A mouse (2102) RYHQRRRDRGHRTSERSLGRYTDVDTGLGTDLSMTTQSGDLPSKDRDQD
      CACNA1A rat (2102) RYHQRRRDRGHRTSERSLGRYTDVDTGLGTDLSMTTQSGDLPSKDRDQD
 CACNA1A_i4human (2150) RHHQRRRDRSHRASERSLGRYTDVDTGLGTDLSMTTQSGDLPSKERDQE
        Consensus (2206) RYHQRRRDRGHR SERSLGRYTDVDTGLGTDLSMTTQSGDLPSKDRDQD
```

Section 47

```
                 (2255) 2255 2260      2270      2280      2290         2303
               cac (1731) EMRERDRLGYGIDMGVTHVQHSYPTLASRRAGIGRRLP-----------
  CACNA1A xenopus (2163) RGRTRDRKHHHHHHHHHHHHHHHPSTERERLEYGRPSS-----------
    CACNA1A mouse (2151) RGRPKDRKHRPHHHHHHHHHHHPPAPDRDRYAQERPDTGRARAREQRWS
      CACNA1A rat (2151) RGRPKDRKHRP-HHHHHHHHHHPPAPDRERYAQERPDTGRARAREQRWS
 CACNA1A_i4human (2199) RGRPKDRKHRQ-HHHHHHHHHHPPPPDKDRYAQERPDHGRARARDQRWS
        Consensus (2255) RGRPKDRKHR  HHHHHHHHHHPPAPDRDRYAQERPDTGRARAREQRWS
```

Section 48

```
                 (2304) 2304  2310      2320      2330      2340       2352
               cac (1769) PTPSKPSTLQLKPTNINFPKLNASPTHTHHSTPHSVHSLPHHRDLLRDP
  CACNA1A xenopus (2201) RSPSTGRDYAPHRQGSSSVDGSPVPSTSGASTPRRGRRQLPQLPTTPRP
    CACNA1A mouse (2200) RSPSEGREHTTHRQGSSSVSGSPAPSTSGTSTPRRGRRQLPQTPCTPRP
      CACNA1A rat (2199) RSPSEGREHATHRQGSSSVSGSPAPSTSGTSTPRRGRRQLPQTPCTPRP
 CACNA1A_i4human (2247) RSPSEGREHMAHRQGSSSVSGSPAPSTSGTSTPRRGRRQLPQTPSTPRP
        Consensus (2304) RSPSEGREH  HRQGSSSVSGSPAPSTSGTSTPRRGRRQLPQTP TPRP
```

```
                                                                          Section 49
                    (2353) 2353      2360      2370      2380      2390          2401
                cac (1818) RDMYYSSRERE--------------------------------------
    CACNA1A xenopus (2250) RVTYSPVVRRPASVCSP------QQQ-----------------------
      CACNA1A mouse (2249) LVSYSPAPRRP--------------------------------------
        CACNA1A rat (2248) LVSYSPAPRRP--------------------------------------
   CACNA1A_i4human (2296) HVSYSPVIRKAGGSGPPQQQQQQQQQQQQQAVARPGRAATSGPRRYPGP
          Consensus (2353)  VSYSPA RRP
                                                                          Section 50
                    (2402) 2402      2410      2420      2430      2440      2450
                cac (1829) ---------R-----------------------------------DRER
    CACNA1A xenopus (2270) SLCRMTTPIQRRYPQTEN--------------------------RPSSN
      CACNA1A mouse (2260) AARRMAGPAAPPGGSPRG---------------------CRRAPRWPAH
        CACNA1A rat (2259) AARRMAGPPAPPGGSPRG---------------------CRRAPRWPAH
   CACNA1A_i4human (2345) TAEPLAGDRPPTGGHSSGRSPRMERRVPGPARSESPRACRHGGARWPAS
          Consensus (2402) AA RMAGP  P GG   G                        A RWPA
                                                                          Section 51
                    (2451) 2451      2460      2470      2480              2499
                cac (1834) LRDRDRDRDRDRLHEYDLRYEYRDRERE-------------------LY
    CACNA1A xenopus (2293) ERRS-VNNDSPRSHRHTRGHCTEVPRAGCRHSG--------------PY
      CACNA1A mouse (2288) APEG----PRP------RGADYTEPDSP-------------------RE
        CACNA1A rat (2287) APEG----PRP------RGADYTEPDSP-------------------RE
   CACNA1A_i4human (2394) GPHVSEGPPGPRHHGYYRGSDYDEADGPGSGGGEEAMAGAYDAPPPVRH
          Consensus (2451) APE     PRPR H Y RGADY EPDSP                   RY
                                                                          Section 52
                    (2500) 2500      2510      2520      2530              2548
                cac (1864) ERERDREREVERERLEYIAPLSFEQALAMGRTGRVLPSPVLNGFKPKS-
    CACNA1A xenopus (2327) RDSSFEDPHPCDQRSSRTAESGGVPPLSPRGRRLPNGYSHGTPRSTKS-
      CACNA1A mouse (2308) PPGGAHDPAPRSPRTPRAAGCA----SPRHGRRLPNGYYAGHGAPRPR-
        CACNA1A rat (2307) PPGGAHEPAPRSPRTPRAAGCA----SPRHGRRLPNGYYAGHGAPRPR-
   CACNA1A_i4human (2443) ASSGATGRSPRTPRASGPACAS----PSRHGRRLPNGYYPAHGLARPRG
          Consensus (2500)    GA DPAPRSPRT R A  A     SRHGRRLPNGYY GHG  RPR
                                                                          Section 53
                    (2549) 2549           2568
                cac (1912) -----GLNPRHSDSDEEDWC
    CACNA1A xenopus (2375) ------RHERYSESEEDDWC
      CACNA1A mouse (2352) -TARRGAHDAYSESE-DDWC
        CACNA1A rat (2351) -TARRGAHDAYSESE-DDWC
   CACNA1A_i4human (2488) PGSRKGLHEPYSESD-DDWC
          Consensus (2549)   ARRG HD YSESE DDWC
```
